# Silica nanoparticles induce lung inflammation in mice via ROS/PARP/TRPM2 signaling-mediated lysosome impairment and autophagy dysfunction

**DOI:** 10.1186/s12989-020-00353-3

**Published:** 2020-06-08

**Authors:** Mingxiang Wang, Jin Li, Shunni Dong, Xiaobo Cai, Aili Simaiti, Xin Yang, Xinqiang Zhu, Jianhong Luo, Lin-Hua Jiang, Binyang Du, Peilin Yu, Wei Yang

**Affiliations:** 1 Department of Toxicology, and Department of Medical Oncology of Second Affiliated Hospital, Zhejiang University School of Medicine, Hangzhou, P. R. China; 2 MOE Key Laboratory of Macromolecular Synthesis and Functionalization, Department of Polymer Science & Engineering, Zhejiang University, Hangzhou, China; 3 Department of Biophysics, and Department of Neurosurgery of the First Affiliated Hospital, Zhejiang University School of Medicine, Hangzhou, P. R. China; 4 The Fourth Affiliated Hospital, Zhejiang University School of Medicine, Yiwu, P. R. China; 5 Institute of Neuroscience, NHC and CAMS Key Laboratory of Medical Neurobiology, Zhejiang University School of Medicine, Hangzhou, P. R. China; 6 Sino-UK Joint Laboratory of Brain Function and Injury and Department of Physiology and Neurobiology, Xinxiang Medical University, Xinxiang, P. R. China; 7 School of Biomedical Sciences, Faculty of Biological Sciences, University of Leeds, Leeds, LS2 9JT UK

**Keywords:** Nanoparticles, Pulmonary inflammation, ROS/PARP/TRPM2 signaling, Lysosomal impairment, Autophagy dysfunction

## Abstract

**Background:**

Wide applications of nanoparticles (NPs) have raised increasing concerns about safety to humans. Oxidative stress and inflammation are extensively investigated as mechanisms for NPs-induced toxicity. Autophagy and lysosomal dysfunction are emerging molecular mechanisms. Inhalation is one of the main pathways of exposing humans to NPs, which has been reported to induce severe pulmonary inflammation. However, the underlying mechanisms and, more specifically, the interplays of above-mentioned mechanisms in NPs-induced pulmonary inflammation are still largely obscure. Considered that NPs exposure in modern society is often unavoidable, it is highly desirable to develop effective strategies that could help to prevent nanomaterials-induced pulmonary inflammation.

**Results:**

Pulmonary inflammation induced by intratracheal instillation of silica nanoparticles (SiNPs) in C57BL/6 mice was prevented by PJ34, a poly (ADP-ribose) polymerase (PARP) inhibitor. In human lung bronchial epithelial (BEAS-2B) cells, exposure to SiNPs reduced cell viability, and induced ROS generation, impairment in lysosome function and autophagic flux. Inhibition of ROS generation, PARP and TRPM2 channel suppressed SiNPs-induced lysosome impairment and autophagy dysfunction and consequent inflammatory responses. Consistently, SiNPs-induced pulmonary inflammation was prevented in TRPM2 deficient mice.

**Conclusion:**

The ROS/PARP/TRPM2 signaling is critical in SiNPs-induced pulmonary inflammation, providing novel mechanistic insights into NPs-induced lung injury. Our study identifies TRPM2 channel as a new target for the development of preventive and therapeutic strategies to mitigate nanomaterials-induced lung inflammation.

**Graphical abstract:**

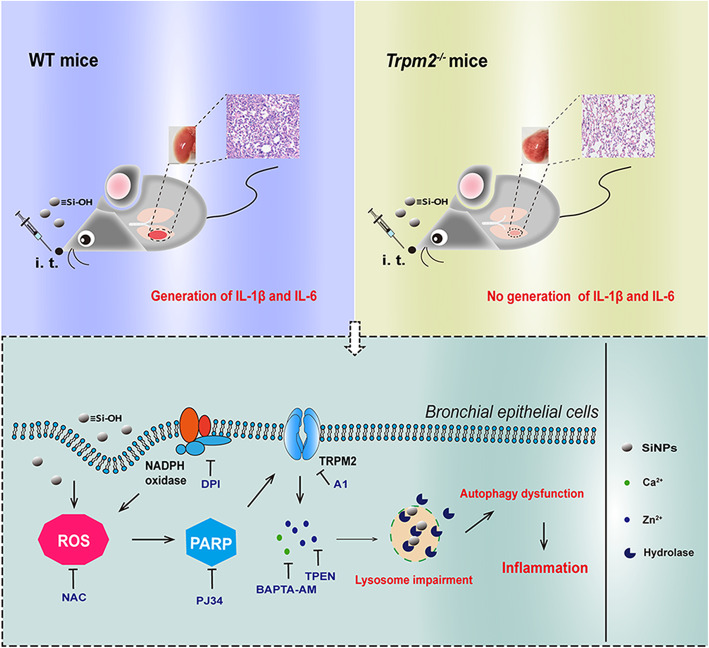

## Background

Nanoparticles (NPs), by definition, have a size of less than 100 nm in at least one of their three dimensions. Growing use of nanomaterials-containing products, and increasing exposure of humans to NPs, have raised concerns about the safety of NPs to humans [1]. Inhalation is one of the main pathways of exposing humans to NPs, and it is generally thought that pulmonary inflammation is a common step [2] that initiates persistent inflammation and induces irreversible lung injury following exposure to NPs [3]. Silica nanoparticles (SiNPs) are amongst the well-accepted and most widely employed nanomaterials, and have applications in cosmetics, food additives, drug delivery, printer toner and paint [3]. Studies demonstrated exposure to SiNPs results in significant increases in the level of pro-inflammatory cytokines in bronchoalveolar lavage fluids (BALFs) and immune cell infiltration in vivo [4]. There is also evidence to support causative association of exposure to SiNPs with pulmonary fibrosis in animal models [5, 6]. Besides, amorphous and crystal silica have been defined by the International Agency for Research on Cancer (IARC) as group 3 (inadequate evidence for carcinogenicity) and group 1 (sufficient evidence for the carcinogenicity to experimental animals and to humans) materials, respectively [7]. Thus, it is important to evaluate pulmonary inflammation induced by exposure to NPs, especially SiNPs, and better understand the underlying mechanisms.

It is well established that elevated reactive oxygen species (ROS) or oxidative stress has a crucial role in SiNPs-induced inflammation [1]. Besides, lysosome impairment and autophagy dysfunction are attracting growing attention for SiNPs-induced damages [8, 9]. Lysosomes contain diverse acidic hydrolases, such as cathepsins, and nanoparticles are reported to accumulate in the lysosomes by endocytosis and subsequently induce cathepsin-dependent inflammation [10]. Recent studies suggest that NPs induce activation of the NLRP3 inflammasome and generation of interleukin-1β (IL-1β) via lysosomal rupture and consequent release of cathepsins [11, 12]. In addition, lysosome functions in the late stage of autophagy to maintain the integrity of autophagy flux, which associates to clearance of damaged organelles and aggregated proteins. SiNPs can induce autophagy mainly via oxidative stress-mediated upregulation of autophagy-related gene expression and differential regulation of the Akt/mTOR signaling [4]. Exposure to SiNPs at high doses or long exposure can also inhibit autophagosome degradation via blockage of autophagic flux [8], leading to disruption of cellular homeostasis [13]. Moreover, accumulating evidence suggests that the autophagy-lysosome pathway plays a key role in regulating systematic and local inflammatory responses [14]. Studies have reported many factors, including ROS generation, inflammation and autophagy dysfunction, are involved in mediating SiNPs-induced toxicity. However, a mechanistic understanding is still lacking and, more specifically, the interplay of ROS generation, lysosome impairment and autophagy dysfunction in SiNPs-induced inflammation. The chemical basis of SiNPs-induced toxicity is still largely obscure. Moreover, lung inflammation is an important target for preventing nanoparticle-induced diseases, and thus a clear understanding of nanoparticle-induced lung inflammation is the prerequisite for developing preventive and therapeutic strategies to alleviate the detrimental consequences of exposure to nanomaterials.

The transient receptor potential melastatin 2 (TRPM2) channel is an ADP-ribose (ADPR)-gated Ca^2+^-permeable non-selective cation channel and is expressed in many cell types [15]. TRPM2 channel is a key oxidative stress sensor, because ROS stimulates production of ADPR via the combined action of PARP and poly (ADP) glycohydrolase (PARG), and thereby activates the TRPM2 channel [16]. TRPM2-mediated Ca^2+^ signaling is known to impact physiologically important processes and functions, including autophagy and cell death [17, 18]. Our previous in vitro study showed that TRPM2 channel plays a role in mediating the detrimental effects of SiNPs on cell viability via promoting NADPH oxidases-mediated ROS production and altering the intracellular Ca^2+^ homeostasis [19]. However, whether such PARP-mediated TRPM2 channel activation is associated with SiNPs-induced pulmonary inflammation in vivo and its specific molecular and cellular mechanisms remain unknown.

In the current study, we demonstrate that intratracheal (i.t.) instillation of SiNPs cause severe lung injury with pulmonary inflammation in mice. We show that exposure to SiNPs impaire lysosome function and autophagy process to induce inflammation in human lung bronchial epithelial (BEAS-2B) cells and further reveal the importance of the ROS/PARP/TRPM2 signaling in mediating SiNPs-induced lysosome impairment, autophagy dysfunction and inflammation. Consistently, transgenic deletion of the TRPM2 expression in mice completely eliminate SiNPs-induced pulmonary inflammation and lung injury, suggesting that TRPM2 channel plays a critical role in this process. We also confirm that the chemical basis of SiNPs-induced toxicity relates to the surface silanol groups, which suggests that modification of these surface groups is a strategy of reducing SiNPs-induced toxicity. Taken together, our study provides novel mechanistic insights into NPs-induced lung injury and identifies the TRPM2 channel as a new target for the development of preventive and therapeutic strategies to nanomaterials-induced lung inflammation.

## Results

### Characterization of SiNPs

**Fig. 1:**
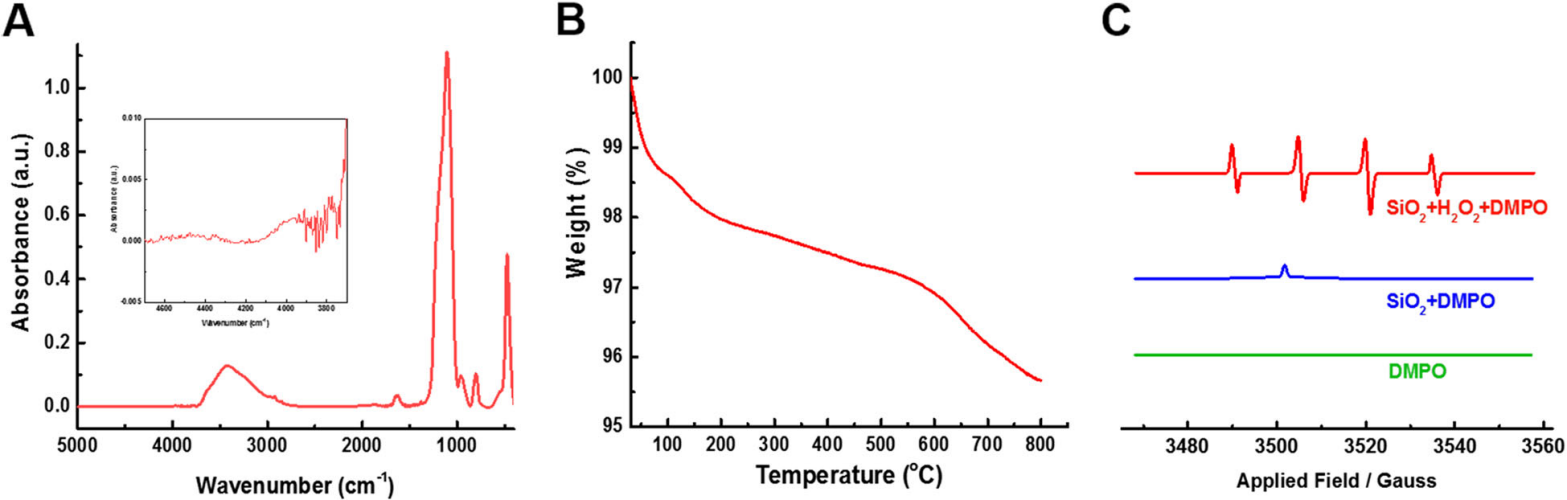
Characterization of SiNPs in suspension. **a** The FTIR spectrum of SiNPs. Insert: The spectrum enlarged in the range of 4700–3700 cm^− 1^. **b** TG analysis showing the weight loss of SiNPs during heating to 800 °C. **C** Derivative EPR data of SiNPs in the presence (red) or absence (blue) of H_2_O_2_, and DMPO without SiNPs as a negative control (green)

SiNPs are well accepted as the most widely used nanoparticles. The morphology and size of commercially available SiNPs were characterized by transmission electron microscopy (TEM). SiNPs dispersed in distilled water by ultrasonication were spherical in shape with an average size of 16.75 ± 3.38 nm (Additional file 1: Figure S1A and B). The surface area of SiNPs, determined by the BET method, was 156 m^2^/g, which is similar with that reported by Zhang et al for fumed silica NPs (Additional file 1: Figure S1C) [20]. The hydrodynamic diameter of SiNPs in water and culture medium, determined by dynamic light scattering (DLS), was 155 ± 10.8 nm and 178 ± 9.61 nm, respectively. The zeta value of SiNPs in distilled water and RPMI-1640 containing 10% FBS was − 25.95 ± 0.63 mV and − 16.81 ± 0.5 mV, respectively, suggesting SiNPs aggregation to some extent (Additional file 1: Figure S1D). Figure 1a showed the FTIR spectrum of SiNPs. A broad absorption peak centered at 3425 cm^− 1^ was assigned to hydrogen-bonded vicinal silanols, and a small narrow peak at 3770 cm^− 1^ in the enlarged spectrum in the range of 4700–3700 cm^− 1^ was attributed to isolated silanols. Clearly, our SiNPs only contained a small fraction of isolated silanols on the surface. Thermogravimetric (TG) analysis indicated about 4.3% of weight loss after heating up to 800 °C in air (Fig. 1b), accompanied with dehydroxylation of the silica surface via condensation reactions of adjacent surface silanoals and, additionally, a decrease in hydrogen-bonded vicinal silanols and an increase in isolated silanols [20]. The electron paramagnetic resonance (EPR) spectrum of SiNPs showed 1:2:2:1 quartet characteristic of DMPO-OH^•^ in the presence but not in the absence of H_2_O_2_, indicating that SiNPs have an ability to generate hydroxyl radicals which can react with H_2_O_2_ to produce OH^•^ according to a Fenton-like reaction (Fig. 1c) [20, 21].

### Exposure to SiNPs induces lung inflammation in mice, depending on PARP activation.

**Fig. 2:**
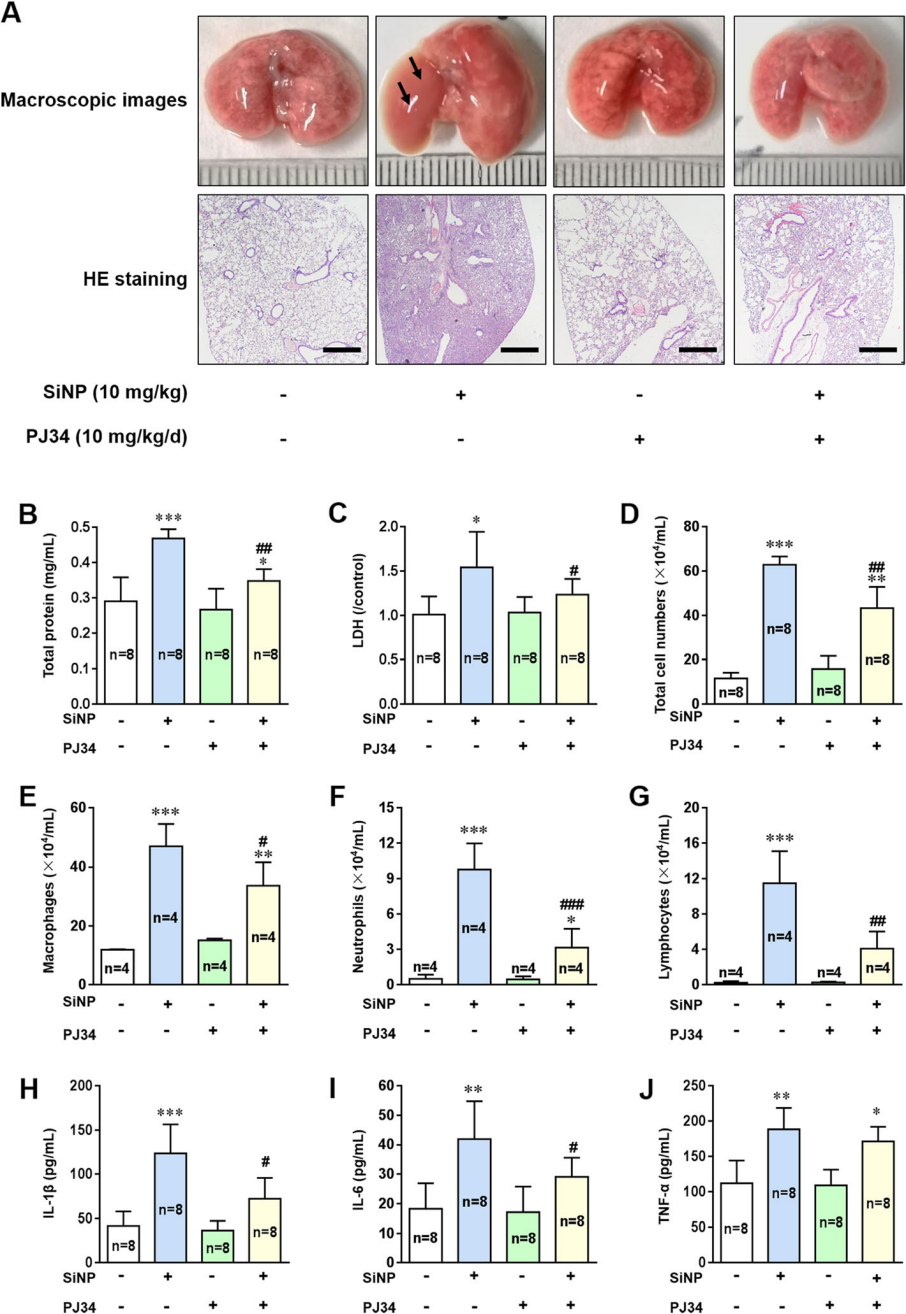
Exposure to SiNPs induces severe lung injury and inflammation in mice, depending on PARP activation. **a** Representative lung histopathology on the 7th day in mice after treated with normal saline or SiNPs (10 mg/kg) in the absence or presence of PJ34 (10 mg/kg/d). Black arrows indicate lung tissue edema. Scale bar = 500 μm. **b**-**g** Total protein concentrations (**b**), LDH release (**c**), total cells numbers (**d**), macrophages numbers (**e**), neutrophils numbers (**f**), and lymphocytes numbers (**g**) in the BALFs of mice determined on the 7th day after treated with normal saline or SiNPs (10 mg/kg) in the absence or presence of PJ34 (10 mg/kg/d). **h**-**j** The concentrations of IL-1β (**h**), IL-6 (**i**), and TNF-α (**j**) in the BALFs of mice under the same conditions as shown for **b**-**g**, as markers of local inflammation were measured by ELISA. *P < 0.05, **P < 0.01, ***P < 0.001 compared with the control group. ^**#**^*P* < 0.05, ^**##**^*P* < 0.01 compared with SiNPs-treated group

We firstly examined lung injury in wild-type (WT) C57BL/6 mice after i.t. instillation of SiNPs. Analysis of morphology and hematoxylin-eosin (H&E) staining indicated that exposure to SiNPs resulted in significant tissue edema and infiltration of inflammatory cells (Fig. 2a). The levels of total protein and lactate dehydrogenase (LDH) in the BALFs, the widely-used markers of airway microvascular permeability and lung injury, respectively, were significantly elevated in SiNPs-exposed mice (Fig. 2b, c). The numbers of total cells, macrophages, neutrophils and lymphocytes were all increased upon SiNPs exposure (Fig. 2d-g). Among these parameters, increased neutrophil infiltration is one of the most sensitive indicators for pulmonary inflammation, and a increase in the number of lymphocytes indicates an immune cell-mediated inflammatory response. We also used enzyme-linked immunosorbent assay (ELISA) to analyze the levels of key pro-inflammatory cytokines, IL-1β, IL-6 and tumor necrosis factor-α (TNF-α) in the BALFs, which were significantly elevated after SiNPs exposure (Fig. 2h-j).

Oxidative stress is the most recognized mechanism mediating nanotoxicity, via acting on different intracellular organelles or activating many signaling pathways. As shown above, EPR analysis indicates that SiNPs have a strong ability to produce hydroxyl radicals. Thus, we further explored the role of oxidative stress in siNPs-induced pulmonary inflammation. PARP is one of the targets by oxidative stress and plays an important role in the DNA repair process. It was reported that PARP activation promotes inflammation by influencing the expression of pro-inflammatory mediators [22], and that administration of PJ34, a PARP inhibitor, markedly decreased lung inflammatory damage induced by bleomycin, an antitumor drug [16]. Consistently, SiNPs-induced adverse effects in the mice were strongly alleviated by treatment with PJ34 (Fig. 2a-i), with an exception that SiNPs-induced generation of TNF-α was not affected (Fig. 2j). Altogether, these in vivo experiments demonstrate that PARP is critically involved in SiNPs-induced generation of IL-1β and IL-6.

### Oxidative stress and PARP activation are involved in SiNPs-induced toxicity and inflammation in BEAS-2B epithelial cells

**Fig. 3:**
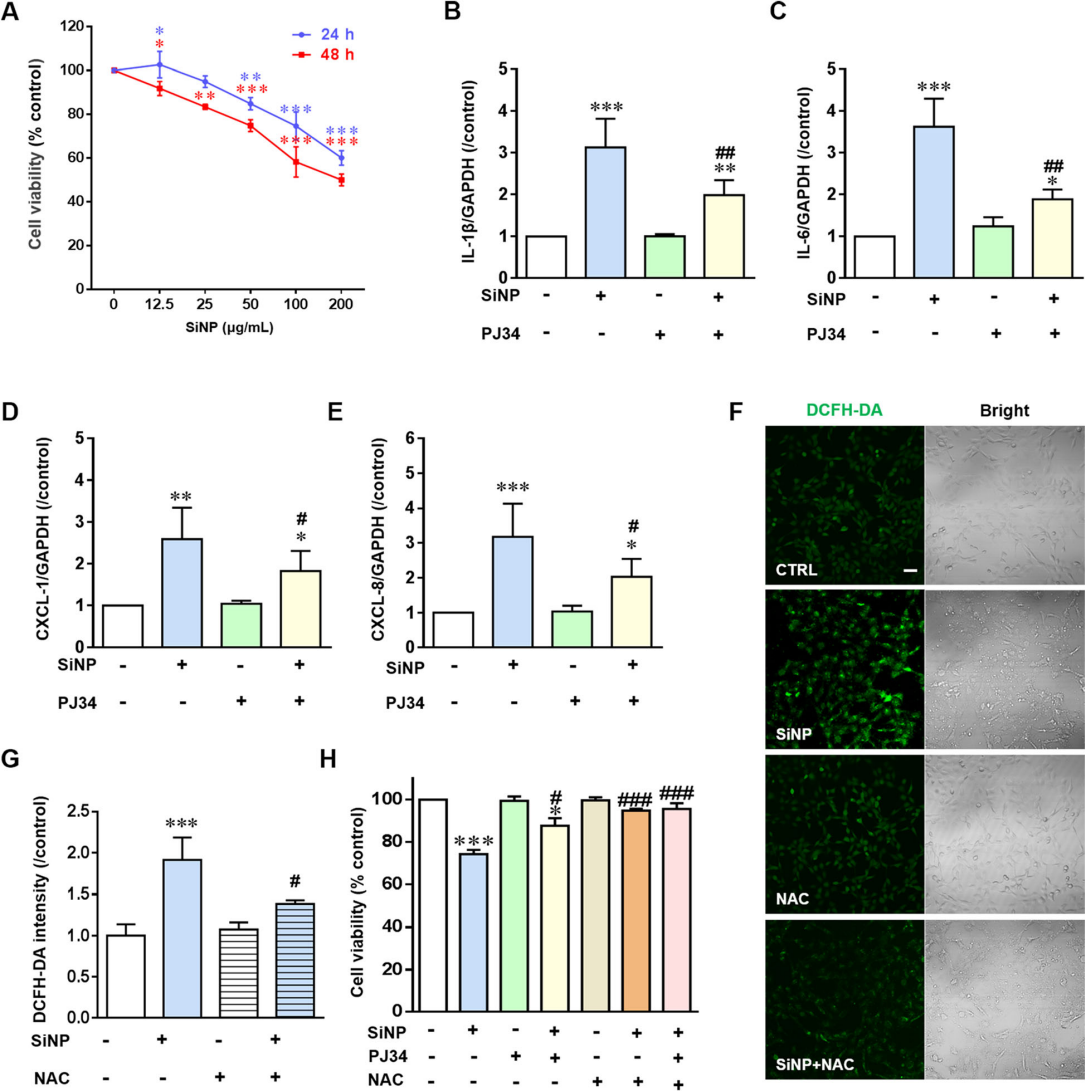
Roles of oxidative stress and PARP activation in SiNPs-induced cytotoxicity in BEAS-2B cells. **a** Cell viability determined using a Cell Counting Kit-8 (CCK-8) after exposure to SiNPs at different doses (0, 12.5, 25, 50, 100 and 200 μg/mL) for 24  and 48 h. **b**-**e** The levels of mRNA expression for IL-1β (**b**), IL-6 (**c**), CXCL-1 (**d**) and CXCL-8 (**e**) in cells under control or after exposure to SiNPs (100 μg/mL) in the absence or presence of PJ34 (10 μM). **f** Representative confocal microscopic images showing DCFH-DA fluorescence intensity in cells under control condition (CTRL), or after treatment with SiNPs (100 μg/mL) for 12 h in the absence or presence of NAC (5 mM), or NAC (5 mM) alone. Scale bar = 50 μm. **g** Mean DCFH-DA fluorescence intensity under indicated conditions, as shown in **f**, from 200 cells analyzed for each condition. **h** Cell viability under control condition and after exposure to SiNPs (100 μg/mL) for 24 h, NAC (5 mM), PJ34 (10 μM) or in combinations. *P < 0.05, **P < 0.01, ***P < 0.001 compared to the control group. ^**#**^*P* < 0.05, ^**##**^*P* < 0.01, ^**###**^*P* < 0.001 compared to SiNPs-treated group

After reaching the respiratory tract, SiNPs firstly contact with epithelial cells and cause damage before they are removed via phagocytosis by macrophages [23, 24]. Human bronchial epithelial BEAS-2B cells and immortalized bone marrow-derived macrophages (iBMDMs) were used to further define the cellular and molecular mechanisms responsible for in vivo pulmonary inflammation and lung injury. Exposure to 12.5–200 μg/mL SiNPs for 24 and 48 h caused significant cell death in a dose- and time-dependent manner in BEAS-2B cells (Fig. 3a). Exposure to 100 μg/mL SiNPs for 24 h was used for further investigations. Under this condition, SiNPs increased the expression levels of IL-1β and IL-6 in BEAS-2B cells (Fig. 3b, c and Additional file 2: Figure S2). Notably, there was no detectable expression of TNF-α, suggesting that SiNPs-induced generation of TNF-α in vivo in mice mainly by macrophages [25]. In addition, generation of CXCL-1 and CXCL-8, which target CXC chemokine receptors on neutrophils, was increased in BEAS-2B cells after SiNPs exposure (Fig. 3d, e and Additional file 2: Figure S2). Considering activation of PARP by oxidative stress, we evaluated SiNPs-induced production of ROS in BEAS-2B cells. Exposure to SiNPs enhanced ROS generation, which was significantly decreased by N-acetyl-L-cysteine (NAC), a ROS scavenger (Fig. 3f and g). Correspondingly, SiNPs-induced reduction in the cell viability was largely rescued by NAC (Fig. 3h). In addition, SiNPs-induced production of cytokines and chemokines, as well as cell death, in BEAS-2B cells were significantly reduced by treatment with PJ34 (Fig. 3b-f and h), demonstrating critical involvement of PARP activation in SiNPs-induced inflammation and cytotoxicity in epithelial cells. Exposure of iBMDMs to 100 μg/mL SiNPs for 24 h induced generation of IL-6 and TNF-α, which however was not sensitive to inhibition by treatment with PJ34 (Additional file 3: Figure S3). Taken together, these results suggest that lung epithelial cell inflammation plays a key role in mediating SiNPs-induced pulmonary inflammation and lung injury. Therefore, BEAS-2B cells were used in subsequent experiments to examine the PARP pathway in mediating SiNPs-induced damage to lung epithelial cells.

**Fig. 4:**
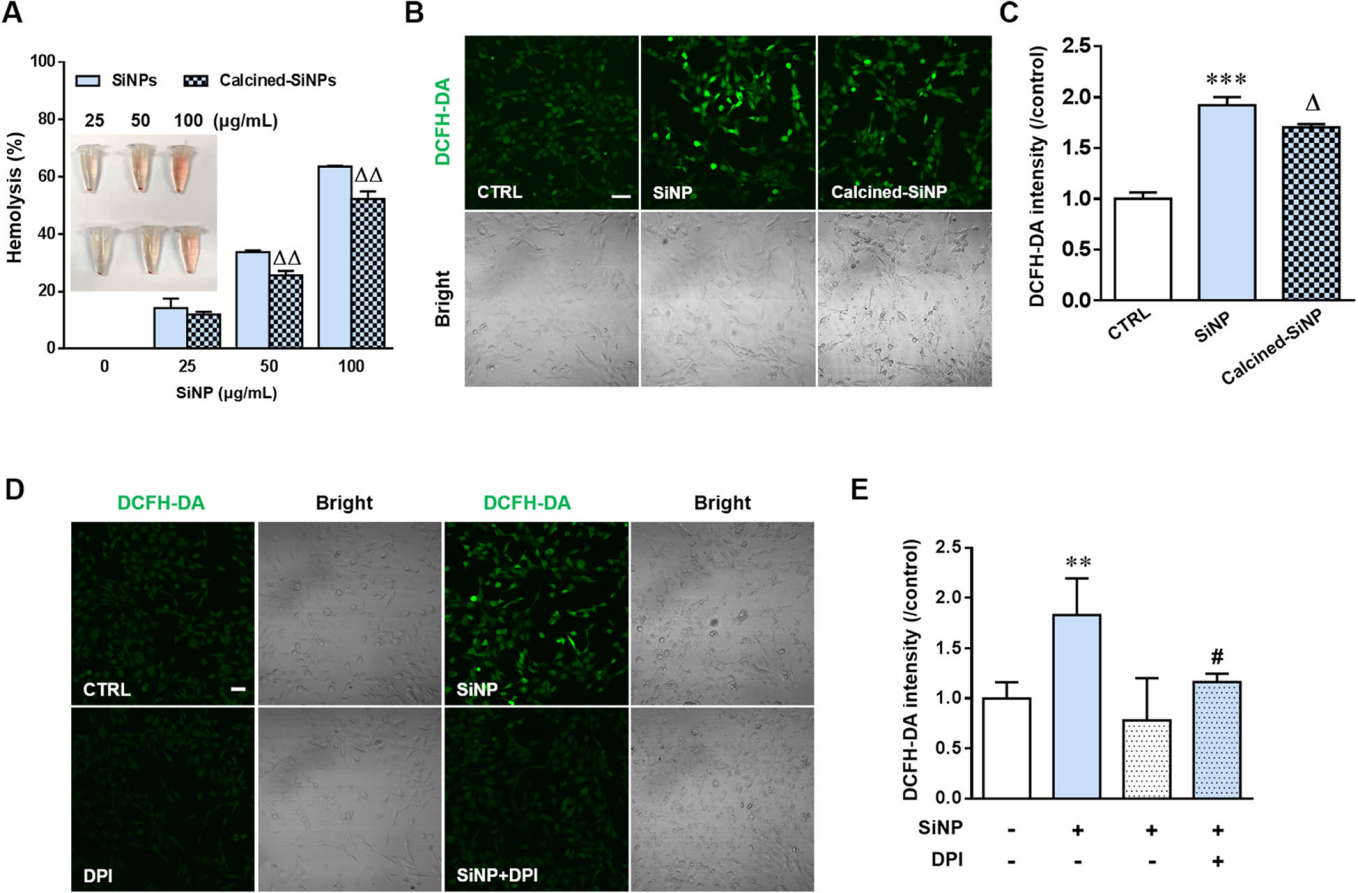
Contribution of surface silanol groups and NADPH oxidases in SiNPs-induced oxidative stress. **a** Hemolysis to mouse RBCs after exposure to 25–100 μg/mL SiNPs (100 μg/mL) or calcined-SiNPs for 3 h at room temperature. The insert represents hemoglobin release indicated by red color in the supernatant. **b** Representative confocal microscopic images showing DCFH-DA fluorescence in BEAS-2B cells under control (CTRL) condition, or after treatment with SiNPs (100 μg/mL) or calcined-SiNPs (100 μg/mL). Scale bar = 50 μm. **c** Mean DCFH-DA fluorescence intensity under indicated conditions as shown in **b**, from 200 cells analyzed for each condition. **d** Representative confocal microscopic images showing DCFH-DA fluorescence in BEAS-2B cells under control (CTRL) condition or after treatment with SiNPs (100 μg/mL) in the absence or presence of DPI (0.1 μM). **e**. Mean DCFH-DA fluorescence intensity under indicated conditions as shown in **d**, from 200 cells analyzed for each condition. **P < 0.01, ***P < 0.001 compared to control group. ^**#**^*P* < 0.05 compared to SiNPs-treated group. ^Δ^*P* < 0.05, ^ΔΔ^*P* < 0.01 compared to calcined-SiNPs at the same concentration

According to the FTIR spectrum, the surface of SiNPs is mainly composed of hydrogen-bonded silanols, which confer the potential to generate hydroxyl radicals (Fig. 1a-c). Thus, we studied the chemical basis of SiNPs-induced generation of ROS and cytotoxicity. Red blood cells (RBCs) are susceptible to oxidative damage, which leads to hemolysis, and the RBC lysis is regarded predictive of cytotoxicity [20, 21]. SiNPs caused dose-dependent RBC lysis (Fig. 4a). Calcination at 600 °C to reduce the total hydroxyl content of silica surface by dehydroxylation significantly reduced hydrogen-bonded silanols on the SiNPs surface [20]. As expected, the ROS level was slightly but significantly decreased in BEAS-2B cells after exposure to calcined-SiNPs (Fig. 4b and c) and hemolysis was also reduced in RBCs incubated with calcined-SiNPs (Fig. 4a). In addition, SiNPs-induced ROS production in BEAS-2B cells was attenuated by treatment with diphenyleneiodonium chloride (DPI), a NADPH oxidases inhibitor (Fig. 4d and e), suggesting that NADPH oxidases is also significantly involved in ROS generation, as reported in our previous study [19].

### SiNPs-induced PARP activation mediates alkalization and reduces the degradation capacity of lysosomes in BEAS-2B cells

**Fig. 5:**
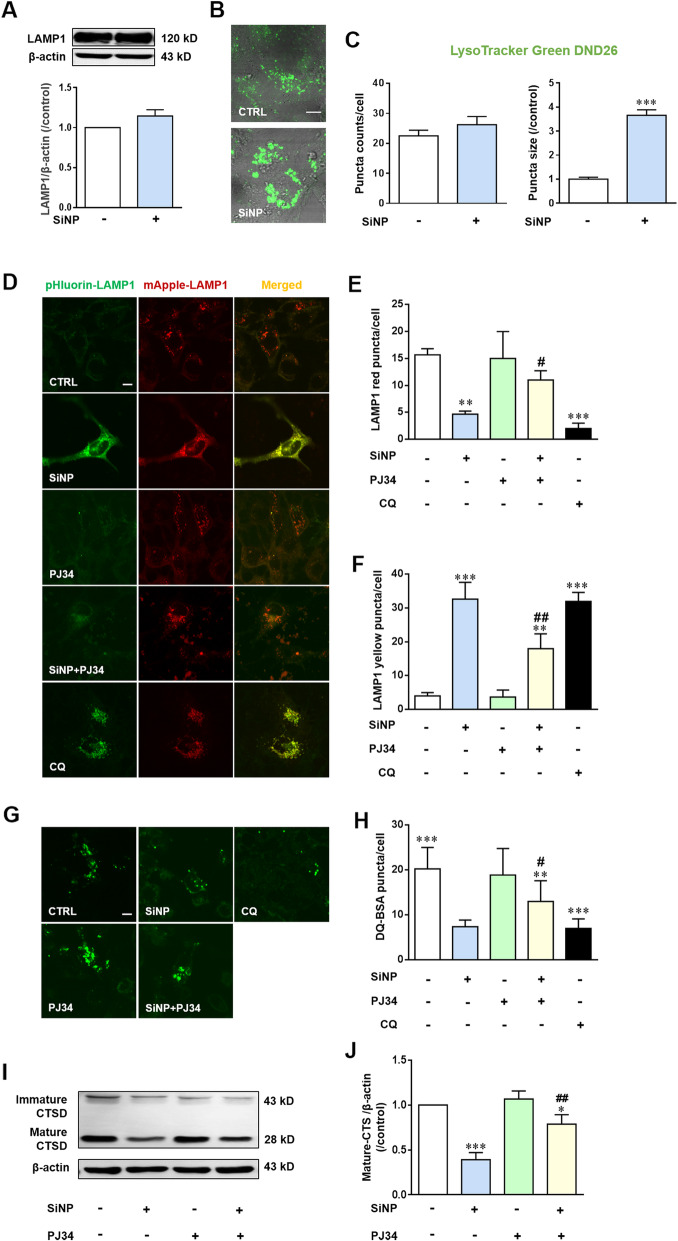
PARP activation mediates SiNPs-induced lysosomal alkalization and reduced degradation capacity in BEAS-2B cells. **a** LAMP1 expression determined by western blotting. **b** Representative images showing LysoTracker Green DND-26 fluorescence in cells under control conditions or after treated with SiNPs (100 μg/mL) for 24 h. **c** The mean number and size of lysosomes in cells as shown in **b**, from 50 cells for each condition. Scale bar = 10 μm. **d** Representative confocal microscopic images showing mApple-LAMP1-pHluorin in cells under control (CTRL) condition or after treatment with SiNPs (100 μg/mL) for 24 h in the absence or presence of PJ34 (10 μM). Cells were incubated with CQ (50 μM) for 3 h as the positive control. Scale bar = 10 μm. **e-f** Mean number of puncta in each cell under the conditions shown in **d**, from 30 cells for each condition. **g** Representative confocal microscopic images showing DQ-BSA analysis of lysosomal proteolytic activity in cells under control condition or after treatment with SiNPs (100 μg/mL) for 24 h in the absence or presence of PJ34 (10 μM). Scale bar = 10 μm. **h** Mean number of puncta in each cell under indicated conditions shown in **g**, from 30 cells for each condition. **i-j** Western blotting analysis of CTSD in BEAS-2B cells under control condition or treatment with SiNPs (100 μg/mL) in the absence or presence of PJ34 (10 μM), from three independent experiments. *P < 0.05, **P < 0.01, ***P < 0.001 compared to the control cells, and ^**#**^*P* < 0.05, ^**##**^*P* < 0.01 compared to cells treated with SiNPs alone

It is well documented that oxidative stress can induce lysosome damage [26]. A previous study reported that NPs imposed remarkable influence on lysosomes [27]. Lysosomal-associated membrane protein 1 (LAMP1) is considered as a lysosome marker and LAMP1 staining is routinely used to visualize lysosomal compartments [28]. Western blotting analysis showed modest but insignificant increase in the LAMP1 expression after SiNPs exposure (Fig. 5a). Fluorescence imaging using LysoTracker Green DND-26, a lysosome-specific probe revealed that the number of lysosomes was slightly increased and the size of lysosomes became significantly larger after uptake of SiNPs (Fig. 5b and c), likely due to defects in lysosome degradation [29]. Given the unique feature of lysosome for its highly acidic lumen (pH 4.5–5.0) that provides optimal conditions for the catalytic function of hydrolytic enzymes, we expressed mApple-LAMP1-pHluorin in BEAS-2B cells to examine the luminal environments in the lysosomes. As a pH-sensitive mutant of GFP, pHluorin is quenched in an acidic environment and emits red fluorescence in functional lysosomes while it produces yellow fluorescence in non-functional lysosomes [30]. As shown in Fig. 5d-f, like exposure to chloroquine (CQ), a lysosome inhibitor, used as a positive control, exposure to SiNPs resulted in significant accumulation of mApple-LAMP1-pHluorin related yellow puncta in BEAS-2B cells, confirming that SiNPs caused alkalinization that may reduce the degradation capacity of lysosomes.

To further investigate the effects of SiNPs on the degradation capacity of lysosomes in BEAS-2B cells, we performed DQ-BSA dequenching analysis. Under normal conditions, non-quenched proteins of DQ-BSA are released through the process of degradation by lysosomes, resulting in bright fluorescence [31]. As shown in Fig. 5g and h, a decrease in DQ-BSA fluorescence was observed in cells after SiNPs exposure. Aspartic protease cathepsin D (CTSD) is one of the most abundant and functionally important lysosomal proteases, and its maturation and release to the cytosol can also reflect the degradation function of lysosomes [32]. Western blotting analysis showed the cytosolic level of mature CTSD was significantly decreased in SiNPs-exposed BEAS-2B cells (Fig. 5i and j). Maturation of cathepsin proteases requires lysosomal acidification and thus alkalization can ultimately impair cathepsin-mediated protein degradation [33], which is in strong agreement with the lysosome impairment observed in our experiment. Finally, SiNPs-induced impairment in lysosome function was significantly mitigated by treatment with PJ34 (Fig. 4d-j), indicating a role of PARP in mediating SiNPs-induced detrimental effect.

### SiNPs-induced PARP-mediated lysosome impairment leads to blockage of autophagic flux

**Fig. 6:**
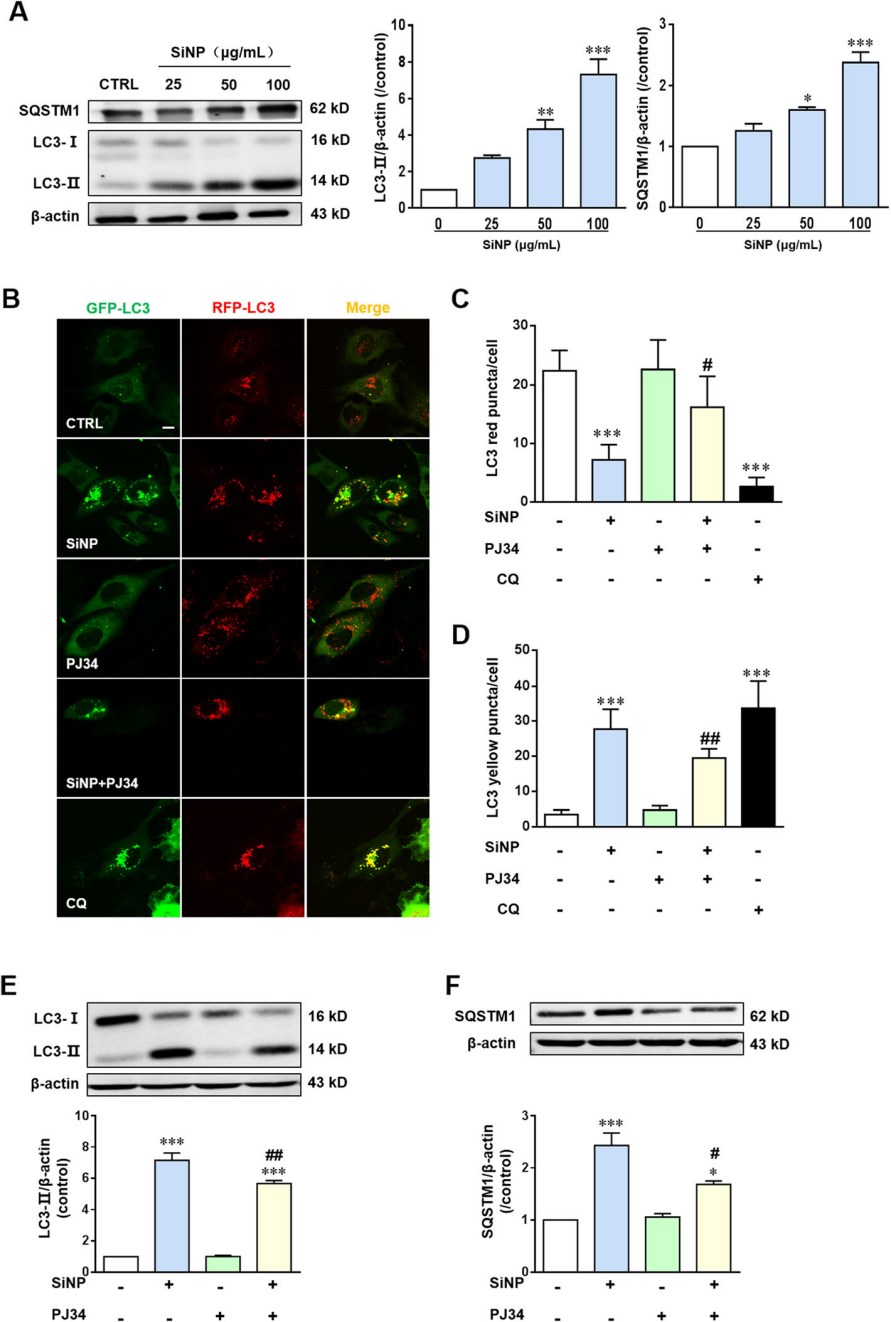
PARP activation mediates SiNPs-induced blockage of autophagic flux in BEAS-2B. **a** Western blotting analysis of the levels of LC3 and SQSTM1 in BEAS-2B cells treated with different concentrations of SiNPs for 24 h. **b** Representative confocal images showing GFP-LC3-RFP in cells treated with SiNPs (100 μg/mL) in the absence or presence of PJ34 (10 μM) at 24 h. Scale bar = 10 μm. **c-d** Mean number of yellow puncta (autophagosomes) and red puncta (autolysosomes) in each cell from 30 cells for each condition. **e-f**. Western blotting analysis of the levels of LC3 (**e**) and SQSTM1 (**f**) in BEAS-2B cells under control condition or after treated with SiNPs (100 μg/mL) in the absence or presence of PJ34 (10 μM), from three independent experiments. *P < 0.05, **P < 0.01, ***P < 0.001 compared to control cells, and ^**#**^*P* < 0.05, ^**##**^*P* < 0.01 compared to cells treated with SiNPs alone

Given the important function of lysosome in autophagy, lysosome impairment inevitably affects the autophagy flux. We therefore evaluated the progression of autophagy in BEAS-2B cells upon SiNPs exposure. Microtubule-associated light chain 3 II (LC3-II) is the most important marker of autophagy [29]. As shown in Fig. 6a, exposure to SiNPs for 24 h caused a significant and dose-dependent elevation in the LC3-II level, indicating an increase in the number of autophagosomes. Such an effect can be associated either with increased autophagosome formation, decreased autophagosomes degradation, or both [34]. To clarify whether the LC3-II accumulation resulted from autophagy activation and/or blockage of autophagic flux, we examined SQSTM1 in SiNPs-exposed BEAS-2B cells. SQSTM1 is a multifunctional protein that binds to LC3 and is degraded within the autolysosome [35], and thus enhanced SQSTM1 level has been regarded as an indicator for blockage of autophagic flux [36]. Our results showed an increase in the SQSTM1 level in BEAS-2B cells after SiNPs exposure (Fig. 6a), suggesting blockage of the autophagic flux. We analyzed transiently expressed GFP-RFP tandem fluorescent-tagged LC3 to further confirm the blockage of autophagic flux. The LC3-II positive autophagosomes are labeled with yellow puncta under normal condition, but are presented as red puncta after fusion with lysosomes to form autolysosomes [23, 32]. Exposure to SiNPs like CQ, significantly promoted accumulation of yellow puncta in BEAS-2B cells (Fig. 6b-d), indicating an increase in the number of autophagosomes. SiNPs-induced autophagy dysfunction was also alleviated by treatment with PJ34 (Fig. 6b-f). Taken together, our results indicate that SiNPs impair autophagic flux in a PARP-dependent manner in BEAS-2B cells.

It is known that induction of lysosomal membrane permeabilization (LMP) leads to inflammation by activating the NLRP3 inflammasome [37, 38]. We were therefore interested in whether activation of the NLRP3 inflammasome contributed in SiNPs-induced inflammatory response [39]. SiNPs-induced increase in the IL-1β level was significantly inhibited by MCC950, a selective NLRP3 inhibitor (Additional file 4: Figure S4), indicating that activation of the NLRP3 inflammasome is involved in SiNPs-induced inflammation. Moreover, it is well known that autophagy plays an essential role in the inflammatory response of lungs to infection and stress [40]. A previous study reported that bismuth NPs-induced acute kidney injury was made worse on one hand by CQ, a blocker of autophagic flux, and on the other was attenuated by rapamycin, an autophagy inducer [41]. We showed that CQ significantly increased the generation of IL-1β and IL-6 in BEAS-2B cells, whereas rapamycin resulted in an opposite effect (Additional file 5: Figure S5), supporting a causative relationship of autophagy with inflammatory response. Taken together, our results suggest that SiNPs-induced blockage of autophagic flux promotes inflammation.

### TRPM2 channel activation mediates SiNPs-induced inflammation via lysosome impairment and blockage of autophagic flux in BEAS-2B cells

**Fig. 7:**
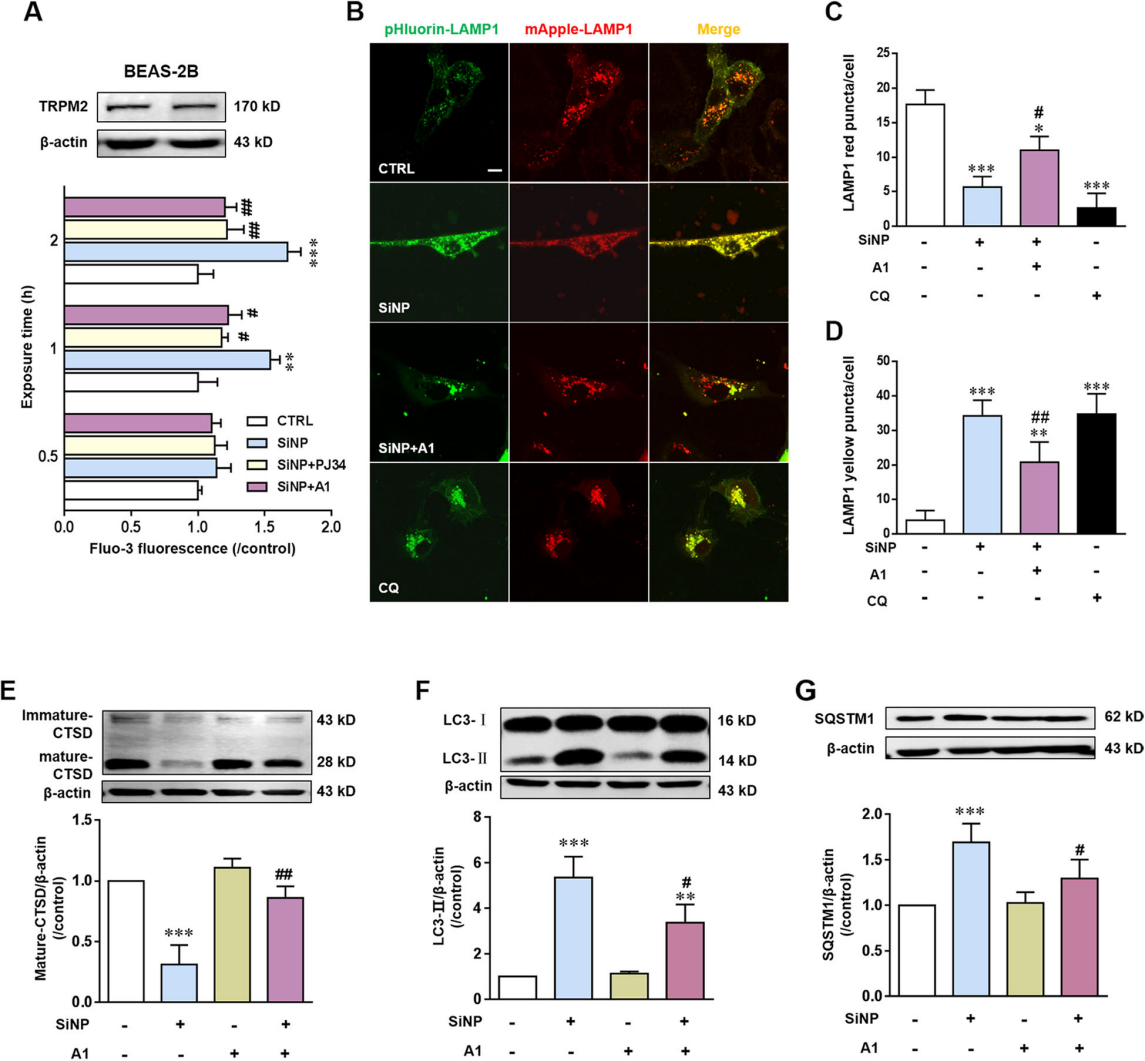
TRPM2 channel activation mediates SiNPs-induced lysosome impairment and blockage of autophagic flux in BEAS-2B cells. **a** Western blotting analysis of TRPM2 expression in cells (top) and single cell imaging analysis using Fluo-3 of intracellular free Ca^2+^ levels in cells treated with SiNPs (100 μg/mL) for 0.5, 1, 2, 3 h in the absence or presence of PJ34 (10 μM) and compound A1 (10 μM) (bottom). **b** Representative confocal microscopic images showing mApple-LAMP1-pHluorin fluorescence in cells under control condition or after treated with SiNPs (100 μg/mL) for 24 h in the absence or presence of compound A1 (10 μM). Scale bar = 10 μm. **c-d** Mean number of puncta in each cell under indicated onditions shown in B, from 30 cells for each condition. **e-g** Western blotting analysis the levels of CTSD (**e**), LC3 (**f**) and SQSTM1 (**g**) in cells under control condition or after treatment with SiNPs (100 μg/mL) in the absence or presence of compound A1 (10 μM), from three independent experiments. *P < 0.05, **P < 0.01, ***P < 0.001 compared to the control cells, and ^**#**^*P* < 0.05, ^**##**^*P* < 0.01 compared to cells treated with SiNPs alone

**Fig. 8:**
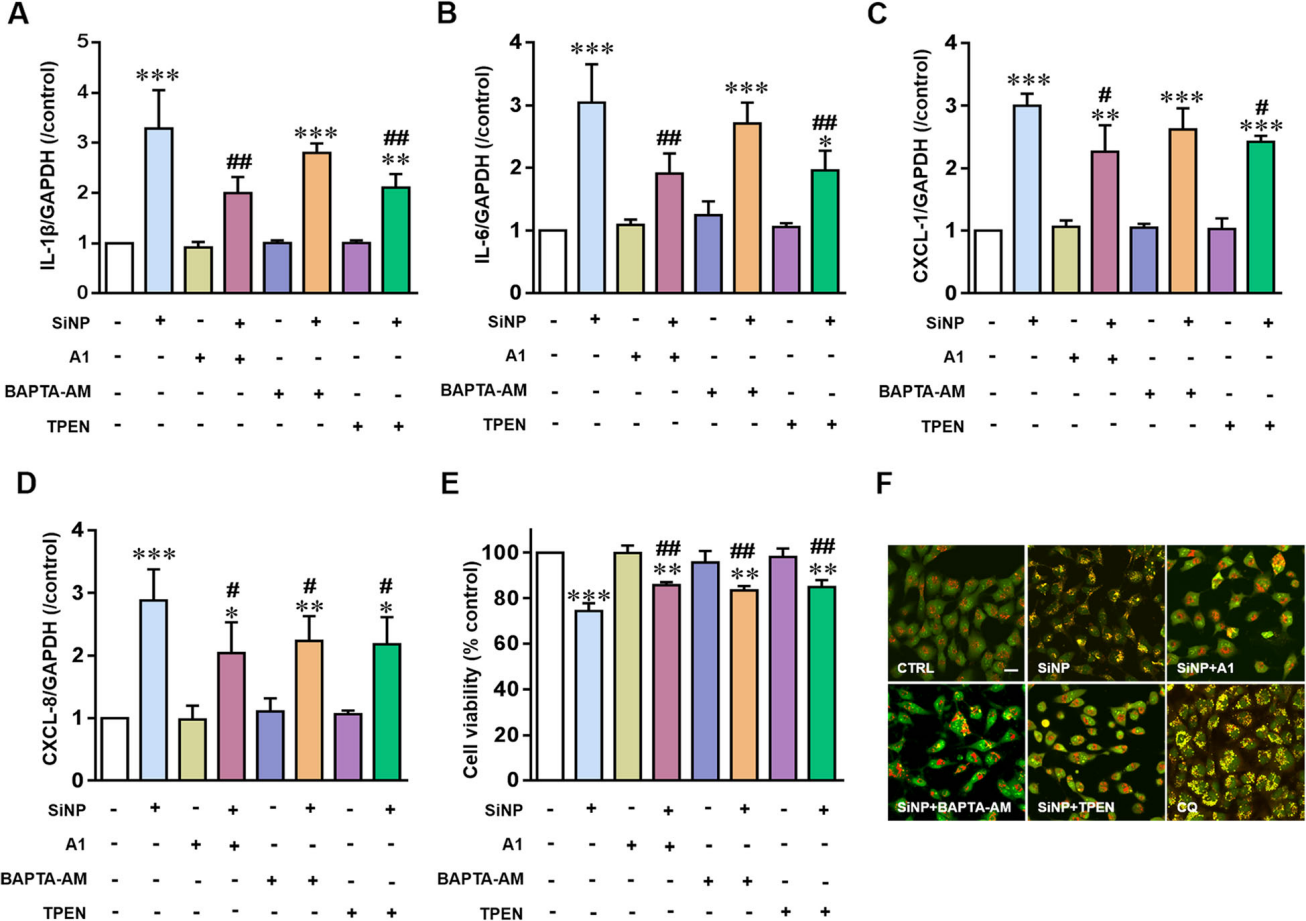
TRPM2-mediated lysosomal and autophagic dysfunction in SiNPs-induced inflammation in BEAS-2B cells. **a-d** Quantitative RT-PCR analysis of the mRNA expression levels for IL-1β (**a**), IL-6 (**b**), CXCL-1 (**c**) and CXCL-8 (**d**) in cells under control condition or after treatment with SiNPs (100 μg/mL) for 24 h with or without prior treatment with compound A1 (10 μM), TPEN (5 μM) or BAPTA-AM (1 μM). **e** Mean cell viability in cells under control condition or after exposure to SiNPs (100 μg/mL) for 24 h with prior treatment with compound A1 (10 μM), TPEN (5 μM) or BAPTA-AM (1 μM), from three independent experiments. **f** Representative confocal microscopic images showing acridine orange (AO) staining in cells under control condition or after exposure to SiNPs (100 μg/mL) for 24 h with or without prior treatment with compound A1 (10 μM), TPEN (5 μM) or BAPTA-AM (1 μM). Cells were incubated with CQ (50 μM) for 3 h as positive control. Scale bar = 20 μm. Data are from *P < 0.05, **P < 0.01, ***P < 0.001 compared to the control cells, and ^**#**^*P* < 0.05, ^**##**^*P* < 0.01 compared to cells treated with SiNPs alone

PARP is known to play a key role in mediating ROS-induced production of intracellular through the combined action of PARP and poly (ADP) glycohydrolase (PARG), the main endogenous agonist of TRPM2 channel [42, 43]. We were prompted to further investigate involvement of the TRPM2 channel in mediating SiNPs-induced epithelial cell inflammation. We started with using compound A1, a TRPM2 channel selective inhibitor developed by our own lab [44]. Western blotting analysis demonstrates TRPM2 protein expression in BEAS-2B (Fig. 7a). Consistently, intracellular free Ca^2+^ concentration was assessed by using Fluo-3, a fluorescent Ca^2+^ indicator, showed that SiNPs-induced intracellular Ca^2+^ increase was inhibited by treatment with compound A1 as well as PJ34 (Fig. 7a). As shown above for PJ34, treatment with compound A1 also attenuated SiNPs-induced reduction in red puncta and increase in yellow puncta in lysosomes in BEAS-2B (Fig. 7b-d). In addition, compound A1 suppressed SiNPs-induced reduction in the level of mature CTSD (Fig. 7e). These results support critical involvement of the TRPM2 channel in SiNPs-induced lysosome impairment. As shown in Fig. 7f and g, compound A1 significantly alleviated SiNPs-induced accumulation of both LC3-II and SQSTM1, suggesting reversal of SiNPs-induced blockage of autophagic flux by inhibiting the TRPM2 channel. We next examined the effects of compound A1 on SiNPs-induced generation of pro-inflammatory cytokines and chemokines, and reduction in the cell viability in BEAS-2B cells. In SiNPs-exposed cells, both elevated generation of IL-1β, IL-6, CXCL-1 and CXCL-8 (Fig. 8a-d and Additional file 2: Figure S2) and cell death (Fig. 8e) were also significantly prevented by treatment with compound A1. Collectively, these results indicate that PARP-dependent TRPM2 channel activation mediates SiNPs-induced inflammation via lysosome impairment and blockage of autophagic flux in BEAS-2B cells.

As a non-selective cation channel, TRPM2 plays a role in ROS-induced cell death through altering intracellular Ca^2+^ and/or Zn^2+^ homeostasis [45, 46]. To define whether TRPM2-dependent alterations in intracellular Ca^2+^ and Zn^2+^ homeostasis in SiNPs-induced lysosome impairment and toxicity in BEAS-2B cells, we determined the effects of BAPTA-AM and TPEN, chelators for Ca^2+^ and Zn^2+^ respectively, in combination with mApple-LAMP1-pHluorin assay and also acridine orange (AO) staining assay. AO emits green fluorescence in the cytosol and nucleus, but red fluorescence when it is accumulated in the acidic compartments [32]. It is anticipated for a decrease in granular red fluorescence with an increase in diffused green fluorescence is anticipated as a result of loss of acidic pH in the lysosomal lumen [47]. Treatment with compound A1 significantly increased red puncta and decreased green fluorescence in cells exposed to SiNPs or CQ, demonstrating the reversal of lysosomal alkalization by blocking the TRPM2 channel (Fig. 8f). Similarly, both BAPTA-AM and TPEN alleviated the SiNPs-induced lysosomal alkalization (Fig. 8f), suggesting that TRPM2-dependent alteration in the intracellular Ca^2+^ and Zn^2+^ homeostasis is critical in SiNPs-induced lysosome impairment. We further evaluated the effects of these two chelators on SiNPs-induced generation of cytokines and chemokines in BEAS-2B cells. As shown in Fig. 7a-d, TPEN significantly reduced SiNPs-induced generation of cytokines and cytokines, whereas BAPTA-AM had a modest but insignificant inhibition. Both chelators alleviated cell death induced by SiNPs (Fig. 8e). These results indicate a critical role for the TRPM2 channel, more specifically, TRPM2-mediated disruption in intracellular Ca^2+^ homeostasis, and particularly Zn^2+^ homeostasis in SiNPs-induced lysosome impairment and inflammation in BEAS-2B cells.

### Genetic deletion of TRPM2 expression eliminates SiNPs-induced pulmonary inflammation and lung injury in C57BL/6 mice

**Fig. 9:**
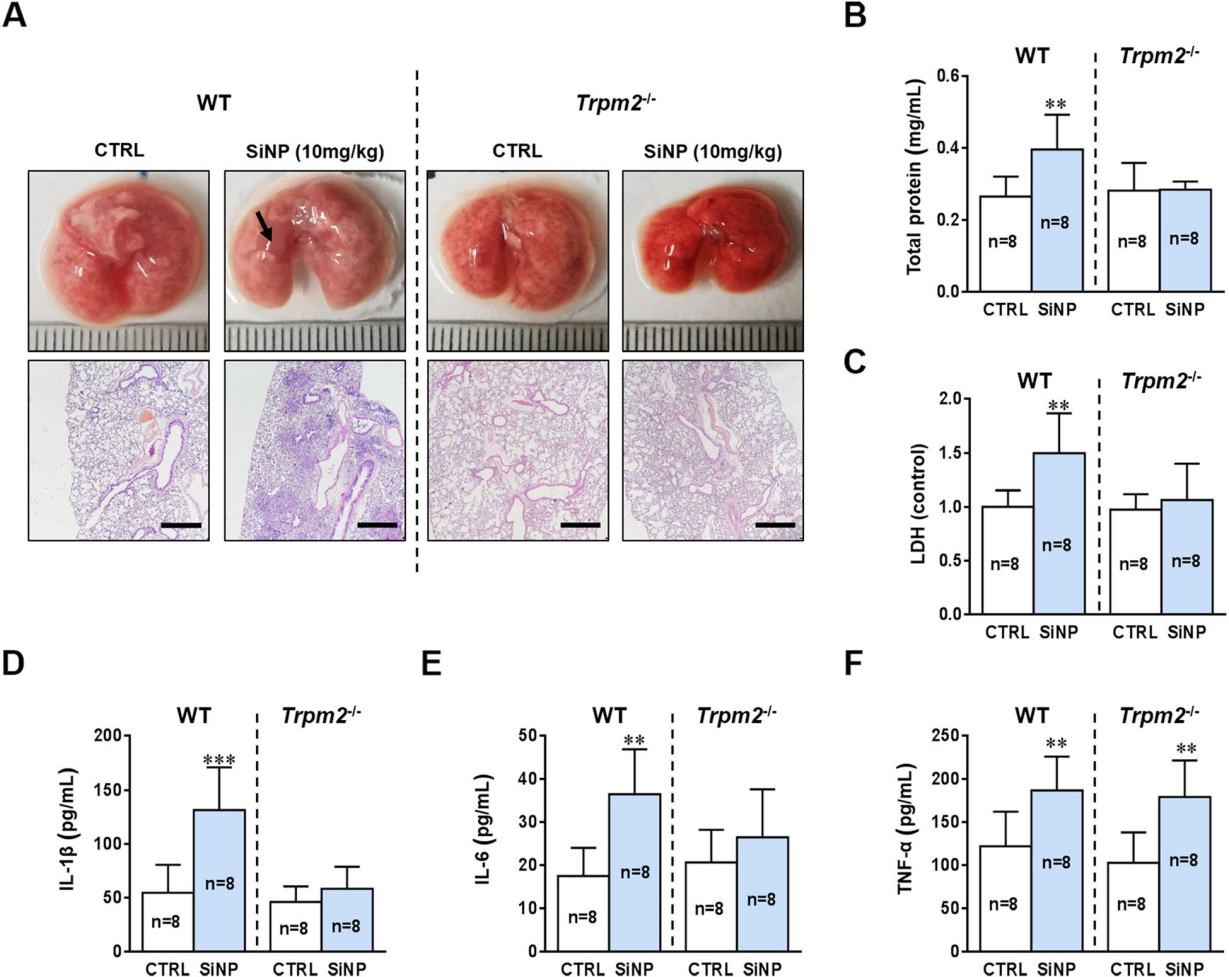
TRPM2 deficiency eliminates SiNPs-induced pulmonary inflammation in C57BL/6 mice. **a** Lung histopathology on the 7th day after treatment with saline or SiNPs (100 μg/mL). Scale bar = 500 μm. **b-c** Total protein concentrations (**b**), and LDH (**c**) in the BALF of *Trpm2*^−/−^ mice on the 7th day after exposure to saline or SiNPs (100 μg/mL). **d-f** Mean concentrations of IL-1β (**d**), IL-6 (**e**), **a**nd TNF-α (**f**) in the BALFs measured by ELISA. The data from the WT mice were displayed on the left side of the dotted line, and the *Trpm2*^−/−^ data on the right side. * *P* < 0.05 compared with the control group

To further verify the significance of our in vitro findings, we examined SiNPs-induced lung injury and pulmonary inflammation in the *Trpm2*^−/−^ C57BL/6 mice. In contrast with the WT mice, the *Trpm2*^−/−^ mice showed no significant lung tissue edema and infiltration of inflammatory cells after SiNPs exposure (Fig. 9a). SiNPs also induced no increase in total proteins and LDH release in the *Trpm2*^−/−^ mice (Fig. 9b and c). Most importantly, SiNPs-induced increase in generation of IL-1β and IL-6 was completely absent in the *Trpm2*^−/−^ mice (Fig. 9d and e). However, the *Trpm2*^−/−^ mice after SiNPs exposure still exhibited generation of TNF-α at a similar level to the WT mice (Fig. 9f), which was in line with no effect of treatment with PJ34 on TNF-α generation under in vivo (Fig. 2j) and in vitro conditions (Additional file 3: Figure S3). Exposure to SiNPs increased the numbers of total cells, macrophages, neutrophils and lymphocytes in the *Trpm2*^−/−^ mice, but SiNPs-induced increases in the *Trpm2*^−/−^ mice were significantly smaller than those in the WT mice after exposure to SiNPs (Additional file 6: Figure S6). Taken together, the results indicate that the TRPM2 channel plays a key role in SiNPs-induced lung injury and pulmonary inflammation.

## Discussion

With SiNPs widely used in food, medicine, agriculture and consumer products, concerns grow regarding their potential risks to human health and the environment. Toxicology of silica materials via inhalation has been reported extensively. While amorphous silica is generally considered safe by the U.S. Federal Drug Administration [48], there is accumulating evidence suggesting the toxicity of amorphous silica nanoparticles [49–51], since Song et al firstly reported that lung toxicity might be induced by long-term exposure to nanoparticles in 2009 [52]. Thus, effective protective methods appear to be important in terms of protecting workers from illness caused by exposure to nanoparticles. Fumed silica as the widely-used commercial SiNPs is one of the two principal classes of amorphous silica nanoparticles. In our study, we first confirmed the toxicological effects of SiNPs on lungs in vivo in mice. The dose range of 2.7 to 15.53 mg/kg SiNPs exposed to mice used in our study is thought close to that exposed to human population [53]. As has been well documented, immune response is a complex process, composed of recruitment of many types of immune cells and subsequent generation of pro-inflammatory cytokines and chemokines at different time courses. A previous study showed that up-regulation of cytokines occurred at an early stage after i.t. instillation with SiNPs and lasted for 1 week, while increases in total cells and proteins in the BALFs were only observed 1 week after exposure [54]. In order to take various factors into account, we examined the mice on the 7th day following i.t. instillation of 10 mg/kg SiNPs. Under such conditions, SiNPs induced severe pulmonary inflammation in WT C57BL/6 mice. Of note, while i.t. instillation has been commonly used experimentally to introduce NPs exposure, it may produce different effects on the lungs, hearts and other organs compared with actual exposure to human population, due to delivery of single high-dose to lungs rather than long-term and repeated exposure to low doses, which should be considered in future studies.

We studied the chemical basis for the ability of SiNPs to induce ROS generation. It has been postulated that the toxicity of amorphous silica is related to the number of silanol groups on the surface [1, 4, 20]. As was reported, fumed SiNPs undergo a progressive surface dihydroxylation leading to a reduction in the total and hydrogen-bonded silanol concentrations over the temperature range from 200 °C to 800 °C [20]. Consistently, calcination of SiNPs to 600 °C significantly reduced the total hydroxyl content and hydrogen-bonded silanols on the surface. This was further confirmed by the reduced ability of calcinated SiNPs to induce ROS production in BEAS-2B cells and hemolysis to RBCs (Fig. 4a-c). Since the surface silanol groups play a more important role in oxidative stress induced by SiNPs, modifications should be considered during synthesis in order to reduce the toxicity of SiNPs. Such a notion is consistent with a recent study showing that the surface silanol content in fumed silica has a key role in, but is not the sole factor, determining the cytotoxicity [55] and providing a starting point for the synthesis of nano-silica materials with less or no toxicity.

We investigated, using BEAS-2B cells, the molecular mechanism underlying SiNPs-induced inflammation in human bronchial epithelial cells, which acts as the first physical barrier to defense against exogenous stimuli. Autophagy dysfunction has been considered to be a potential toxic effect of nanoparticles that results in cytotoxicity and inflammation [56]. Here, our studies showed that SiNPs exposure activated the TRPM2 channel through ROS/PARP pathway, which induced lysosome impairment and subsequent blockage of autophagic flux in epithelial cells. So far, most studies focus on the effects of nanoparticles on autophagy induction rather than the process of autophagy degradation [4]. Yu et al. showed that SiNPs increased formation of autophagosomes and autolysosomes in HepG2 cells [57]. Duan et al. reported that SiNPs induced autophagic activity in endothelial cells, accompanied by disturbing NO/NOS activity and inducing inflammatory response, via inhibiting the PI3K/Akt/mTOR signaling pathway [58]. In recent years, attention has been drawn to the effects of SiNPs on autophagic flux. Wang et al. reported that SiNPs induced autophagosome accumulation in hepatocytes via activating the EIF2AK3 and ATF6 UPR pathways without effect on lysosomal function [59], whereas Schütz et al. showed accumulation of SiNPs in HeLa cells caused defective autophagic flux and lysosomal dysfunction, albeit without effect on lysosomal acidification or intralysosomal hydrolase activity [9]. A separate study showed that exposure to zinc oxide-NPs impaired autophagic flux and acute lung injury, but it did not investigate the effects on lysosome function [60]. Two in vitro studies reported that gold NPs [29] and carbon nanotubes [61] induced lysosome impairment and subsequent blockage of autophagic flux, but the associations with cytotoxicity and inflammatory responses were not established. Therefore, our finding provide new insights into the underlying mechanisms by showing ROS production, lysosome impairment and autophagy dysfunction and their close interplays in SiNPs-induced inflammatory responses and toxicity to lungs. Such novel findings are helpful to better understand the cytotoxicity and pulmonary fibrosis as a result of exposure to other nanomaterials.

More significantly, our study shows the TRPM2 channel as a key mechanism for SiNPs-induced pulmonary inflammation and lung injury. TRPM2 channel is expressed in various types of immune cells such as dendritic cells [62, 63] and neutrophils [64, 65], and epithelial cells [16, 66, 67] as well as macrophages and is important in inflammation [16, 68, 69]. Our in vivo evidence indicates that TRPM2 channel is critically involved in SiNPs-induced lung injury and increase in the number of mcarophages, neutrophils and lymphocytes. Moreover, SiNPs-induced release of IL-1β and IL-6 also strongly depends on TRPM2 channel, suggesting critical involvement of the TRPM2 channel in SiNPs-induced inflammation. It is well known that epithelial cells, macrophages and neutrophils can mediate generation of cytokines. Our in vitro evidence supports TRPM2 channel is required for SiNPs-induced generation of cytokines in BEAS-2B rather than in iBMDMs. Interestingly, TRPM2 plays a critical role in SiNPs-induced generation of CXCL-1 and CXCL-8 in BEAS-2B cells. CXCL-1 and CXCL-8 play a key role in neutrophil infiltration and subsequent induction of macrophages to a pro-inflammatory phenotype and robust IL-1β release [70]. Our results suggest that TRPM2 channel in epithelial cell is critical for SiNPs-induced neutrophil infiltration and macrophage migration, which might contribute to the SiNPs-induced generation of IL-1β, IL-6 and TNF-α. However, our results suggest that TRPM2 in macrophages may be not critically involved in SiNPs-induced inflammation. Previous studies had reported that TRPM2 played a key role in inflammation. For example, TRPM2 deficiency suppressed exacerbation of inflammation in mouse model of colitis [68]. In contrast, there is evidence to suggest that IL-6 release was increased in TRPM2-deficient mice in sepsis model [71]. Herein, the role of TRPM2 channel in inflammation seems to depend on specific physiological and pathological conditions.

Our study further shows that the TRPM2 channel mediates SiNPs-induced lysosome impairment and inflammation by disrupting the intracellular Ca^2+^, particularly Zn^2+^ homeostasis. A previous study reported that exposure of pancreatic β-cells to high glucose induced oxidative stress and subsequent TRPM2 channel-mediated Ca^2+^ influx to cause lysosomal permeabilization and redistribution of lysosomal Zn^2+^ to mitochondria [72]. Li et al [73] have proposed a positive feedback mechanism for ROS-induced neuronal death, by which TRPM2 channel activation triggers lysosomal dysfunction, lysosomal Zn^2+^ release, mitochondrial Zn^2+^ accumulation, mitochondrial dysfunction and ROS generation. Such effects were suppressed by treatment with PJ34, TRPM2 inhibitors or TPEN. A similar mechanism may operate in mediating SiNPs-induced lysosome impairment in BEAS-2B cells. However, these effects resulting from exposure to SiNPs were not strongly attenuated but not fully abolished by inhibiting PARP or TRPM2 channel, suggesting involvement of other mechanisms, which needs further study. In addition, it is interesting to note that TRPM2 knockout completely eliminated SiNPs-induced generation of IL-1β and IL-6, differing from in vitro experiments where inhibition of the TRPM2 channel with compound A1 only partially alleviated SiNPs-induced generation of cytokines. Such a difference likely reflects the extent of TRPM2 channel inhibition by pharmacological and genetic means. Compound A1 at high concentrations was cytotoxic, hindering us from testing anti-inflammatory effects of compound A1 at increased concentrations. In addition, TRPM2 knockout blocked not only the function of TRPM2 channel in the ROS/PARP/TRPM2 signaling, but also other possible inflammatory effects mediated by the TRPM2 channel. Taken together, this study shows the importance of the ROS/PARP/TRPM2 signaling pathway in SiNPs-induced pulmonary inflammation. Targeting the TRPM2 channel may provide an intervention strategy to mitigate the toxic effects of exposure to nanomaterials.

## Conclusions

**Fig. 10:**
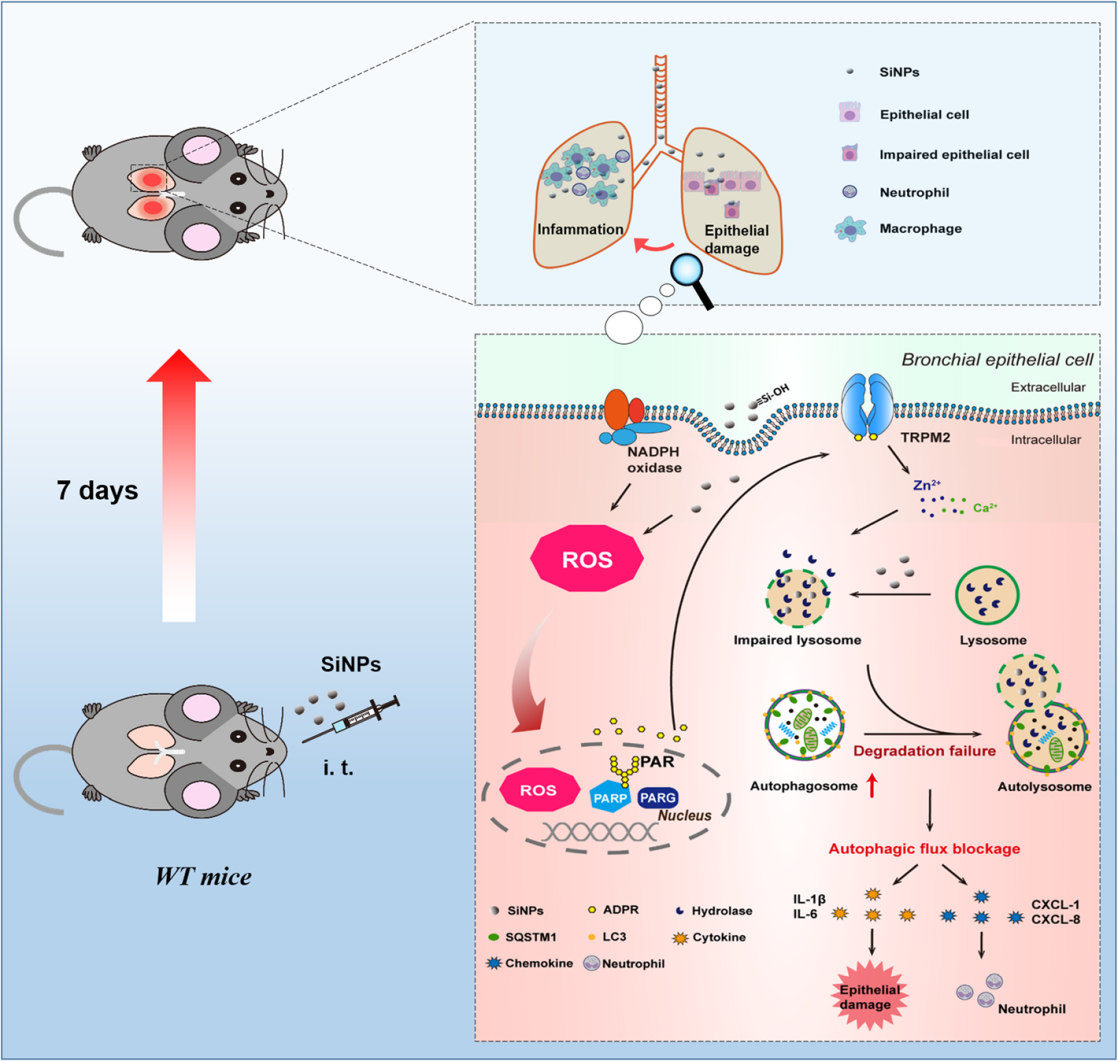
Schematic illustration of the ROS/PARP/TRPM2 signaling pathway that mediates SiNPs-induced pulmonary inflammation in mice. SiNPs are ingested through intratracheal exposure and, upon endocytosis into bronchial epithelial cells, induce ROS production, which activates the TRPM2 channel through the PARP-mediated generation of ADPR in the nucleus. TRPM2 channel activation in turn increases intracellular zinc and calcium ions to impair the degradation function of lysosomes. Lysosomal dysfunction blocks autophagic flux that triggers inflammation by generating cytokines, IL-1β and IL-6, and also chemokines, CXCL-1 and CXCL-8, which are critical for neutrophil recruitment

In summary, for the first time, we show that the ROS/PARP/TRPM2 signaling is critical in SiNPs-induced pulmonary inflammation, and that the surface silanol groups of SiNPs, especially the hydrogen-bonded silanols, play an important role in the generation of hydroxyl radicals. SiNPs exposure induces lysosome impairment and subsequent blockage of autophagic flux, via ROS generation, TRPM2 channel activation and TRPM2-mediated alteration in intracellular Zn^2+^ and Ca^2+^ homeostasis. As a result, dysfunctional autophagy triggers generation of pro-inflammatory mediators leading to pulmonary inflammation. A brief conceptual diagram is presented in Fig. 10. These findings may offer a new strategy to alleviate nanomaterials-induced toxicity by blocking the TRPM2 channel.

## Methods

### Characterization of silica nanoparticle (SiNPs)

Silicon dioxide nanoparticles used in this study were obtained from Sigma-Aldrich ((particle size: 10–20 nm, 99.5% purity) St Louis, MO, USA) and was dispersed in distilled water as stock, and sonicated before use (160 W, 20 kHz, 5 min; JY 92-IIN; Scientz, Ningbo, China)**.** The morphology of SiNPs was examined by transmission electron microscopy (Tecnai G2 Spirit 120Kv, FEI, Czech). The hydrodynamic diameter and zeta potential of SiNPs in distilled water and RPMI-1640 culture media with 10% fetal bovine serum (FBS) were measured using dynamic light scattering (Nano-S90, Malvern Instruments, UK) and Zetasizer Nano Series (Malvern Instruments, UK), respectively. To confirm the endotoxin content of SiNPs, Bioendo EC Endotoxin Test Kit (EC325454S, Bioendo, Xiamen, China) was used and 0.0748 ± 0.0111 EU/mL was detected in SiNPs at 100 μg/mL.

The surface area of SiNPs was measured by the BET method from N_2_ sorption isotherm, which was recorded by a 24-h vacuum outgassing process at 120 °C on automatic specific surface and aperture analysis instrument (AUTOSORB-IQ2-MP). The weight changes in dehydration and dihydroxylation of SiNPs were determined on a Q50 thermogravimeter (TA instruments, USA), in which 3 mg of SiNPs was gradually heated to 800 °C in air with a heating rate of 10 °C/min. Fourier transform infrared (FTIR) spectrum was recorded on a Bruker Vector 22 spectrometer with KBr pellet. The ability of SiNPs to generate free radicals was examined using a Bruker A300 electron paramagnetic (EPR) instrument. The X-band (9.8 GHz) spectrum was recorded at room temperature using 5,5-dimethyl-pirroline-*N*-oxide (DMPO) as the spin-trapping molecule. SiNPs (5% in w:v) were dispersed in the mixture of equal volume of DMPO aqueous solution (12.5 mg/mL) and H_2_O_2_ (30%).

### Hemolysis assay

Mouse blood samples were obtained from three healthy mice and stabilized with EDTA. After the serum was removed by centrifugation and suction, red blood cells (RBCs) were washed several times with sterile isotonic PBS until the supernatant was transparent. Following the last wash, RBCs were diluted to 1/10 of the original volume with sterile isotonic PBS. 300 μL of RBC suspension was mixed with 1200 μL of isotonic PBS as a negative control, 1200 μL of nanopure water as a positive control, or 1200 μL of SiNPs or calcined-SiNPs at 600 °C with concentrations from 25 to 100 μg/mL. The mixtures were vortexed and left to rest for 3 h at room temperature. The samples were centrifuged at 10000 x *g* at 4 °C for 3 min and the absorbance of the supernatants at 541 nm was measured using a microplate reader (Tecan Infinite M200, Switzerland). Hemolysis was calculated by the difference in the absorption between SiNPs-treatment sample and negative control, as percentage of the difference in the absorption between positive and negative controls.

### Animals and exposure

Eight-week-old C57BL/6 WT and *Trpm2*^−/−^ male mice were used in this study. The WT mice were purchased from Shanghai SLAC Laboratory Animal Co. Ltd. (Shanghai, China), and the *Trpm2*^−/−^ mice were bred in Zhejiang University after introduced from University of Leeds, where the transgenic mice were generated. Mouse embryonic clones carrying the mutated allele lacking exons 17 and 18 of the *trpm2* gene and a neomycin resistance and thymidine kinase selection cassette were injected into C57BL/6-derived blastocysts. Homozygous *Trpm2*^−/−^ mice were obtained by several rounds of cross-breeding and validated by PCR of genomic DNA [74]. All mice in this study were group-housed in cages with regular rodent chow and mineral water provided ad libitum under standard breeding conditions with a 12-h reversed light/dark cycle and ~ 22 °C. All animal experiments were performed strictly in accordance with the ethical guidelines by the Ethics Committee of Laboratory Animal Care and Welfare, Zhejiang University School of Medicine. The WT mice were randomized into four groups (*n* = 8 for each group): control group, SiNPs-treatment group, PJ34-treatment group, and SiNPs/PJ34-treatment group. The *Trpm2*^−/−^ mice were divided into two groups (n = 8 for each group): control group and SiNPs-treatment group. All mice in the SiNPs-treatment groups were anesthetized using isoflurane and SiNPs suspended in normal saline (6 mg/mL) were intratracheally instilled slowly with a dosage of 10 mg/kg body weight (40 μL). PJ34 (HY-13688A, MCE, USA) was dissolved in normal saline and administered to mice intraperitoneally 1 h with a dosage of 10 mg/kg body weight before i.t. instillation with SiNPs and were daily injected for another 7 days without SiNPs exposure. The control groups received normal saline.

### Bronchoalveolar lavage fluids (BALFs) and cell counts

All mice were sacrificed on the 7th day after exposure to SiNPs. The trachea was clearly visualized after separating the tissues and skin, and a 20-gauge cannula was inserted into the trachea. The lungs were lavaged with 0.8 mL of ice-cold PBS for twice and 1.2 mL of BALFs collected from each mouse. Then BALFs were centrifuged at 500x *g* at 4 °C for 15 min. The supernatant was transferred to a new tube and frozen for subsequent analysis. The cell pellet was suspended in 500 μL of PBS and the total cell counts were counted using hemocytometer. Counting different cells (macrophages, neutrophils and lymphocytes) was evaluated on a cytospin slide stained with Wright-Giemsa dyes (BA-4017, Baso, Zhuhai, China) and 300 cells per mouse were examined under a light microscope.

### Analysis of BALFs

The concentration of total proteins in the BALFs was measured using Enhanced BCA Protein Assay Kit (P0009, Beyotime, Shanghai, China). The levels of IL-1β, IL-6, TNF-α in the BALFs were determined using ELISA Kit (ELM-IL1β-1/ELM-IL-6-1/ELM-TNFα-1, Raybiotech, GA, USA), and the amount of LDH released in the BALFs was assessed using a LDH Cytotoxicity Assay Kit (C0017, Beyotime, Shanghai, China), according to the manufacturers’ instructions.

### Histological examination

Mice were euthanized under ether anesthesia on the 7th day after SiNPs exposure. All mice were placed on an iced table. The right lung was stored in liquid nitrogen, and the left lung was fixed in 4% paraformaldehyde for 48 h at 4 °C, embedded in paraffin, and serially cut into 5-μm sections. After dewaxing, the sections selected from each mouse were stained with hematoxylin and eosin (H&E) and evaluated the histology of the lung tissues under a light microscope (Olympus BX53, Tokyo, Japan).

### Cell culture

The non-tumorigenic human bronchial epithelial cells (Ad12-SV40 immortalized) BEAS-2B were kindly provided by Prof. Xiangwei Gao (Institute of Environmental Medicine, Zhejiang University School of Medicine, China) and cultured in Roswell Park Memorial Institute medium (RPMI-1640, 31,800, Gibco, USA) with 10% FBS, 100 IU/mL penicillin and 100 μg/mL streptomycin in a 5% CO_2_ humidified atmosphere at 37 °C. Cells were seeded at a density of 5 × 10^3^, 1.5 × 10^4^, 3 × 10^5^ cells/well in 96-well, 24-well and 6-well plates, respectively, to conduct subsequent different experiments. Treatment with SiNPs was performed as described previously. Briefly, BEAS-2B cells were seeded overnight at a 60–70% confluence and treated with SiNPs or with an equal volume of PBS. The immortalized bone marrow derived macrophages (iBMDMs) derived from C57BL/6 mice were kindly provided by Prof. Feng Shao (National Institute of Biological Sciences, China) [75, 76]. iBMDM cells were cultured in Dulbecco’s Modified Eagle Medium (DMEM, 12800, Gibco, USA) with 10% FBS, 100 IU/mL penicillin and 100 μg/mL streptomycin in a 5% CO_2_ humidified atmosphere at 37 °C. Both of two types of cells were exposed to SiNPs following pretreatment with various inhibitors and chelators for 30 min.

### Cell viability assay

The viability of BEAS-2B cells was determined using Cell Counting Kit-8 (C0043, Beyotime, Shanghai, China) according to the manufacturer’s instructions. Briefly, cells were seeded in 96-well plates at a density of 5 × 10^3^ cells/well and treated with SiNPs (12.5, 25, 50 and 100 μg/mL) with or without PJ34 (10 μM), NAC (5 mM, A7250, Sigma, USA), compound A1 (10 μM), TPEN (5 μM, P4413, Sigma, USA) and BAPTA-AM (1 μM, A1076, Sigma, USA) for 24 or 48 h. Cells were washed twice with PBS and CCK-8 was added to each well. After further incubated for 1.5 h, the absorbance at 450 nm was evaluated using a microplate reader (Tecan Infinite M200, Switzerland).

### Detection of intracellular ROS

ROS was detected using DCFH-DA staining and fluorescence imaging. BEAS-2B cells were grown on glass-bottom dishes (Cellvis, CA, USA) to 70% confluence, and treated with SiNPs (100 μg/mL) for 12 h in the presence or absence of NAC (5 mM), and SiNPs-calcined (100 μg/mL) at 600 °C. Thirty minutes prior to imaging, cells were fed with fetal bovine serum free RPMI-1640 loaded with DCFH-DA (10 μM, S0033, Beyotime, Shanghai, China) in dark and kept in a CO_2_ incubator at 37 °C. Cells were washed twice with HBSS (#14025092, Gibco, USA) and visualized under an Olympus FV1000 confocal microscope. Data were analyzed using ImageJ software. DCFH-DA intensity was analyzed by integrated intensity across the whole image divided by total cell number in the same mage using ImageJ.

### Quantitative RT-PCR

Total RNA was isolated using a RNAiso Plus kit (9109, Takara, Shiga, Japan) and was reverse-transcribed to cDNA using a PrimeScript™ RT reagent Kit with gDNA Eraser (RR047A, Takara, Shiga, Japan), according to the manufacturer’s instructions. The cDNA were stored at − 80 °C until used. Quantitative PCR was performed using a SYBR Premix Ex Taq™ II Kit (RR820A, Takara, Shiga, Japan) and a 7500 Fast Real-Time PCR System (Applied Biosystems, Thermo Fisher Scientific, USA). Relative mRNA expression was calculated using the 2^−ΔΔCT^ method, and normalized to GAPDH. The primers for IL-1β, IL-6, CXCL-1, CXCL-8 and GAPDH used are listed as follows:

IL-1β-human.

Forward: 5′-AGCTGATGGCCCTAAACAGA-3′.

Reverse: 5′-TGGTGGTCGGAGATTCGTAG-3′.

IL-1β-mouse.

Forward: 5′-TGGTGGTCGGAGATTCGTAG-3′.

Reverse: 5′-CTAATGGGAACGTCACACACCA-3′.

IL-6-human.

Forward: 5′-CCACTCACCTCTTCAGAACG-3′.

Reverse: 5′-CATCTTTGGAAGGTTCAGGTTG-3′.

IL-6-mouse.

Forward: 5′-CATCTTTGGAAGGTTCAGGTTG-3′.

Reverse: 5′-TTGTATCTCTGGAAGTTTCAGATTGTT-3′.

TNF-α-human.

Forward: 5′-TAGCCCATGTTGTAGCAAACC-3′.

Reverse: 5′-ATGAGGTACAGGCCCTCTGAT-3′.

TNF-α-mouse.

Forward: 5′-GCCACCACGCTCTTCTGTCTAC-3′.

Reverse: 5′-GCCACCACGCTCTTCTGTCTAC-3′.

CXCL-1-human.

Forward: 5′- CCAAACCGAAGTCATAGCCAC-3′.

Reverse: 5′- TGCTCCCCTTGTTCAGTATCT-3′.

CXCL-8-human.

Forward: 5′- ACTGAGAGTGATTGAGAGTGGAC-3′.

Reverse: 5′- AACCCTCTGCACCCAGTTTTC-3′.

GAPDH-human.

Forward: 5′-ACAGTCCATGCCATCACTG-3′.

Reverse: 5′-AGTAGAGGCAGGGATGATG-3′.

GAPDH-mouse.

Forward: 5′-AGTAGAGGCAGGGATGATG-3′.

Reverse: 5′-AGTAGAGGCAGGGATGATG-3′.

### Elisa

The levels of IL-1β, IL-6, CXCL-1 and CXCL-8 in culture medium were assessed using ELISA kits (ELH-IL1β-1/ELH-IL-6-1/ELH-GROα-1/ELH-IL8–1, Raybiotech, GA, USA) according to the manufacturer’s recommendations. In the experiments testing the effects of inhibitors, cells were treated with PJ34 (10 μM), compound A1 (10 μM) or MCC950 (100 nM) 30 min before and during subsequent exposure to SiNPs (100 μg/ml) for 24 h.

### Western blotting

BEAS-2B cell were lysed in RIPA lysis buffer (P0013B, Beyotime, Shanghai, China) containing PMSF (1 mM). After centrifugation at 20000 x *g* for 10 min, the supernatant was collected and its concentration was determined using an Enhanced BCA Protein Assay Kit (P0009, Beyotime, Shanghai, China). Forty micrograms of proteins were separated by SDS-PAGE using 8% gels for detecting TRPM2 and LAMP1, or 12% gels for SQSTM1, LC3B and Cathepsin-D. β-actin was used as the protein loading control. Proteins were transferred onto polyvinylidene fluoride (PVDF) membranes (Immobilon-P, Millipore, MA, USA), and blocked in 5% skim milk in Tris-buffered saline containing 0.1% Tween 20 (v/v) (TBST) for 2 h at room temperature. The membranes were incubated with primary antibodies for TRPM2 (ab96785, 1:1000 dilution, Abcam, USA), LAMP1 (#9091, 1:1000 dilution, CST, USA), SQSTM1 (PM045, 1:1000 dilution, MBL, Japan), LC3B (#12741, 1:1000 dilution, CST, USA), Cathepsin-D (ab75852, 1:1000 dilution, Abcam, USA), and β-actin (A5316, 1:5000 dilution, Sigma, USA) overnight at 4 °C. After subjected to three 5-min washes in TBST, the membranes were incubated with secondary antibodies conjugated with various fluorophores (926–68,020, anti-mouse; 926–32,211, anti-rabbit; 1:5000 dilution, LI-COR, USA), and proteins were visualized and analyzed using a LI-COR Odyssey Infrared Fluorescent System.

### LysoTracker green staining

BEAS-2B cells were seeded on glass-bottom dishes. After exposure to SiNPs (100 μg/mL) for 24 h, the cells were washed twice with HBSS and incubated for 30 min with 1 mL of prewarmed HBSS containing LysoTracker Green DND-26 dye (200 nM, L7526, Invitrogen, USA) [27]. After washing with HBSS, cells were viewed and imaged using a confocal fluorescence microscope (Olympus FV1000, Tokyo, Japan). The number and size of lysosomes in each cell was analyzed using ImageJ.

### Plasmids and transient transfection

The mApple-LAMP1-pHluorin-N-8 construct was a gift from Dr. Michael Davidson (#54918, Addgene, USA). BEAS-2B cells at 60% confluence were transiently transfected with mApple-LAMP1-pHluorin or RFP-LC3-GFP plasmids using Lipofectamine 2000 (11,668,019, Invitrogen, USA) according to the manufacturer’s instructions. Transfected cells were cultured in glass-bottom dishes. For lysosome assay, BEAS-2B cells expressing mApple-LAMP1-pHluorin were treated with PJ34 or compound A1 (both at 10 μM), 30 min prior to and during exposure to SiNPs (100 μg/mL) for 24 h, and imaged under a confocal fluorescence microscope. For autophagy assay, BEAS-2B cells expressing RFP-LC3-GFP were treated with SiNPs (100 μg/mL) for 24 h in the presence or absence of PJ34 (10 μM). Cells treated with 50 μM CQ (C6628, Sigma, USA) for 3 h were used as a positive control. Images from at least three different fields per dish were captured using a confocal microscope. Cells were examined using ImageJ for lysosome function and autophagic flux. Normal lysosomes show red puncta, and impaired lysosomes show yellow puncta, and the ratio of red to yellow puncta in each cell was derived to indicate lysosome function. Autolysosomes show red puncta, and autophagosomes show yellow puncta. Autophagic flux was assessed by the ratio of autophagosomes to autolysosomes in each cell.

### Acridine orange staining

Acridine orange (AO), a lysosomotropic weak base that accumulates in intracellular acidic compartments due to proton trapping, was used to measure the degradation function of lysosomes [77]. It is a metachromatic lysosomotropic dye that fluoresces red when accumulated at high concentrations in the lysosomes and green at low concentrations in the cytosol and the nucleus. Cells seeded on glass-bottom dishes were exposed to SiNPs (100 μg/mL) in the absence or presence of compound A1 (10 μM), TPEN (5 μM), or BAPTA-AM (1 μM) for 24 h, and stained with 5 μg/mL AO (A6014, Sigma, USA) at 37 °C for 30 min. After washing twice with PBS, cells were imaged under a confocal fluorescence microscope (Olympus FV1000, Tokyo, Japan) with excitation at 488 nm (green fluorescence) or 546 nm (red fluorescence).

### Analysis of lysosomal degradation capacity

DQ-BSA, a BSA derivative with green fluorescence quenched after cleavage by proteolytic enzyme, was utilized to detect lysosomal degradation capacity. Cells seeded on glass-bottom dishes were incubated with 10 μg/mL DQ-BSA-Green (D12050, Invitrogn, USA) for 12 h (37 °C, 5% CO_2_) and washed with PBS before exposed to SiNPs (100 μg/mL) in the absence or presence of PJ34 or compound A1 (both at 10 μM). Images were captured using a confocal microscope with excitation at 488 nm. The degradation capacity was estimated by measuring the green fluorescence intensity using imageJ.

### Intracellular calcium measurement

The effect of SiNPs on intracellular free Ca^2+^ concentration was determined by using Fluo-3/AM (F1242, Invitrogen, USA) following the manufacturer’s instructions. In brief, BEAS-2B cells were seeded in 96-well plates (3603, Costar, USA) and incubated with Fluo-3/AM (3.5 μM) at 37 °C for 1 h. The cells were washed with HBSS and incubated with PJ34 and compound A1 (both at 10 μM) 30 min prior to addition of SiNPs (100 μg/mL). Fluorescence was measured at 0.5, 1, 2, 3 h in a SynergyMx M5 microplate reader (Molecular Devices, USA) with excitation at 488 nm and emission at 525 nm. The intensity of samples was normalized to that of the control. Each group had 3 replicate wells and all procedures were performed in the dark.

### Statistical analysis

Data were expressed as mean ± standard deviation (SD) from at least three independent experiments. All the data shown in this study showed a normal distribution and therefore unpaired Student’s *t*-test was used to compare two groups and one-way ANOVA with post hoc Bonferroni test to compare more than two groups, using SPSS 22.0. A *p*-value of less than 0.05 was considered statistically significant. All graphics were prepared using Prism 6 (GraphPad Software, La Jolla, CA, USA).

## Supplementary information

**Additional file 1 : Figure S1** Characterization of SiNPs in suspension. A) The representative morphologies of SiNPs shown using transmission electron microscopy (TEM). Scale bar = 20 nm. B) Size-distribution histograms obtained using Nano Measurer software. C) The surface area of SiNPs determined by the BET method. D) The hydrodynamic size, polydispersity index and zeta potential of SiNPs determined using DLS and Zetasizer Nano Series, respectively.

**Additional file 2 : Figure S2** Inhibition of PARP and TRPM2 channel reduces SiNPs-induced cytokines and chemokines genernation in BEAS-2B cells. IL-1β (A), IL-6 (B), CXCL-1 (C) and CXCL-8 (D). Cells were incubated with SiNPs (100 μg/mL) in the absence or presence of PJ34 or compound A1 (both at 10 μM). Data are presented as mean ± SD from three independent experiments. * *P* < 0.05, ** *P* < 0.01 compared with the control group. ^**#**^*P* < 0.05, ^**##**^*P* < 0.01, ^**###**^*P* < 0.001 compared with SiNPs-treated group.

**Additional file 3 : Figure S3** Treatment with PJ34 has no effect on SiNPs-induced cytokines and chemokines generation in iBMDM cells. IL-1β (A), IL-6 (B), and TNF-α (C). Cells were exposed to SiNPs (100 μg/mL) in the absence or presence of PJ34 (10 μM). Data are presented as mean ± SD from three independent experiments. ** *P* < 0.01 compared with the control group.

**Additional file 4 : Figure S4**. Inhibition of NLRP3 inflammasome attenuates SiNPs-induced inflammation in BEAS-2B cells. Cells were co-incubated with SiNPs (100 μg/mL) for 24 h in the absence or presence of MCC950 (100 nM). Data are presented as mean ± SD from three independent experiments. * *P* < 0.05, *** *P* < 0.001 compared to the control group. ^**##**^*P* < 0.01 compared to SiNPs-treated group.

**Additional file 5 : Figure S5**. Autophagic flux regulates inflammatory responses in BEAS-2B cells. IL-1β (A) and IL-6 (B). BEAS-2B cells were treated with 50 μM chloroquine (CQ) or 100 nM rapamycin (RAPA) for 3 h. Data are presented as mean ± SD from three independent experiments. * *P* < 0.05, ** *P* < 0.01, *** *P* < 0.001 compared to the control group.

**Additional file 6 : Figure S6**. Analysis of inflammatory cells in the BALFs of WT and *Trpm2*^*−/−*^ mice. The counts of total cells (A), macrophages (B), neutrophils (C), lymphocytes (D) in the BALFs from WT and *Trpm2*^−/−^ mice after i.t instillation of SiNPs (10 mg/kg). Data are mean ± SD from 8 mice. *P < 0.05, **P < 0.01, ***P < 0.001 compared with the control group. ^**#**^*P* < 0.05, ^**##**^*P* < 0.01 compared with the WT mice.

## Data Availability

The datasets used and/or analyzed during this study are available from the corresponding authors on reasonable request.

## Abbreviations

NPs: Nanoparticles
SiNPs: Silica nanoparticles
PARP: poly (ADP-ribose) polymerase
ROS: Reactive oxygen species
TRPM2: Transient receptor potential melastatin 2
BALFs: Bronchoalveolar lavage fluids
DLS: Dynamic light scattering
TEM: Transmission electron microscopy
FTIR: Fourier transform infrared
LDH: Lactate dehydrogenase
IL: Interleukin
ELISA: Enzyme-linked immunosorbent assay
TNF-α: Tumor necrosis factor-α
H&E: hematoxylin and eosin
RBCs: Red blood cells
NAC: N-acetyl-L-cysteine
LMP: Lysosomal membrane permeabilization
LAMP1: Lysosomal-associated membrane protein 1
CQ: Chloroquine
RAPA: Rapamycin
CTSD: Cathepsin-D
AO: Acridine orange
RT-PCR: Reversa transcription-polymerase chain reaction
TG: Thermogravimetric
EPR: Electron paramagnetic resonance
IARC: International Agency for Research on Cancer
