# Supplementary figures and images for: Silica nanoparticles induce lung inflammation in mice via ROS/PARP/TRPM2 signaling-mediated lysosome impairment and autophagy dysfunction
Source: Part Fibre Toxicol. 2020 Jun 8;17:23. doi: 10.1186/s12989-020-00353-3 (PMC7281956; doi:10.1186/s12989-020-00353-3)

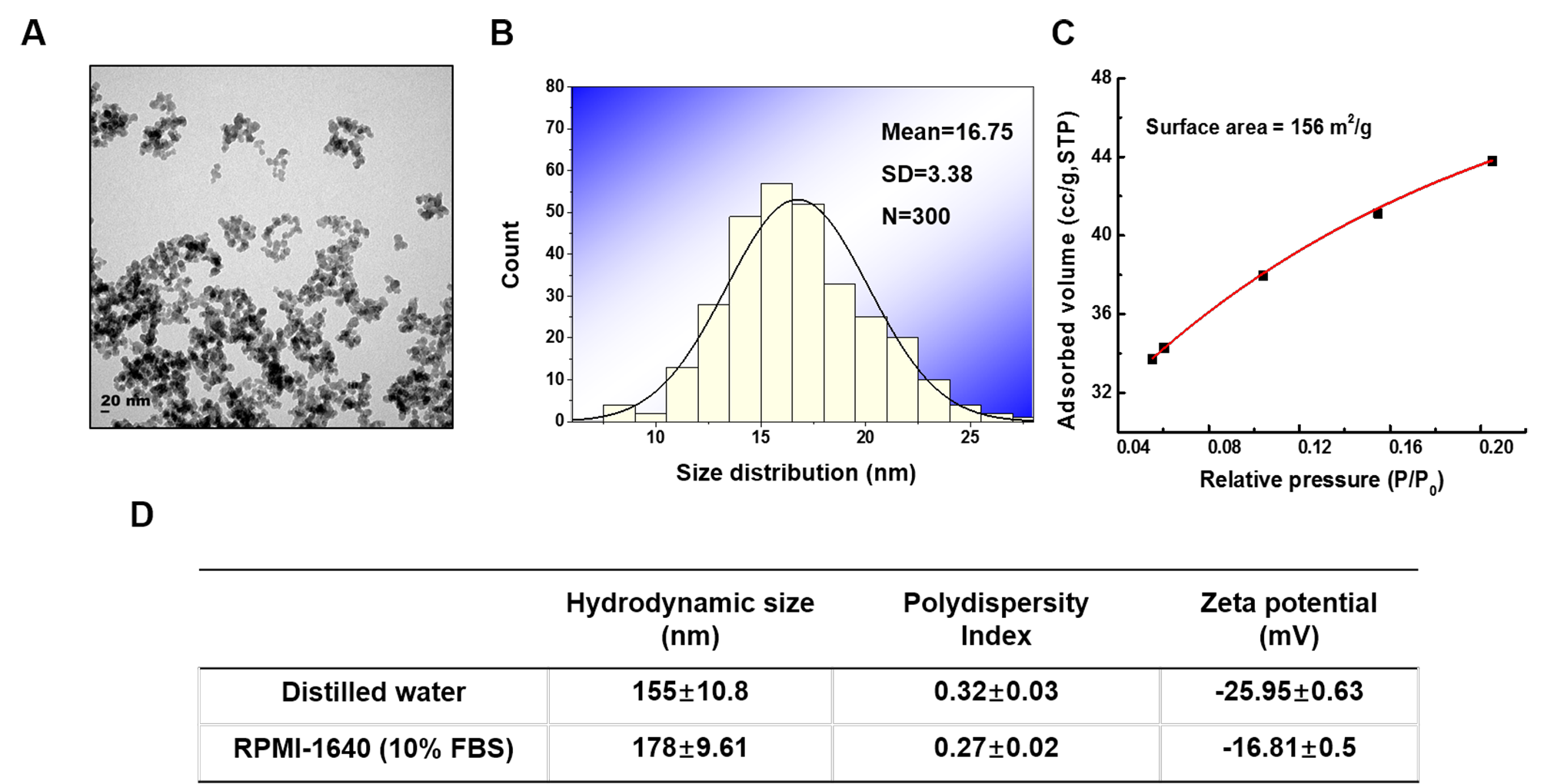

Supplement: Supplementary file 1 — Additional file 1 : Figure S1 Characterization of SiNPs in suspension. A) The representative morphologies of SiNPs shown using transmission electron microscopy (TEM). Scale bar = 20 nm. B) Size-distribution histograms obtained using Nano Measurer software. C) The surface area of SiNPs determined by the BET method. D) The hydrodynamic size, polydispersity index and zeta potential of SiNPs determined using DLS and Zetasizer Nano Series, respectively. [file 12989_2020_353_MOESM1_ESM.tif]

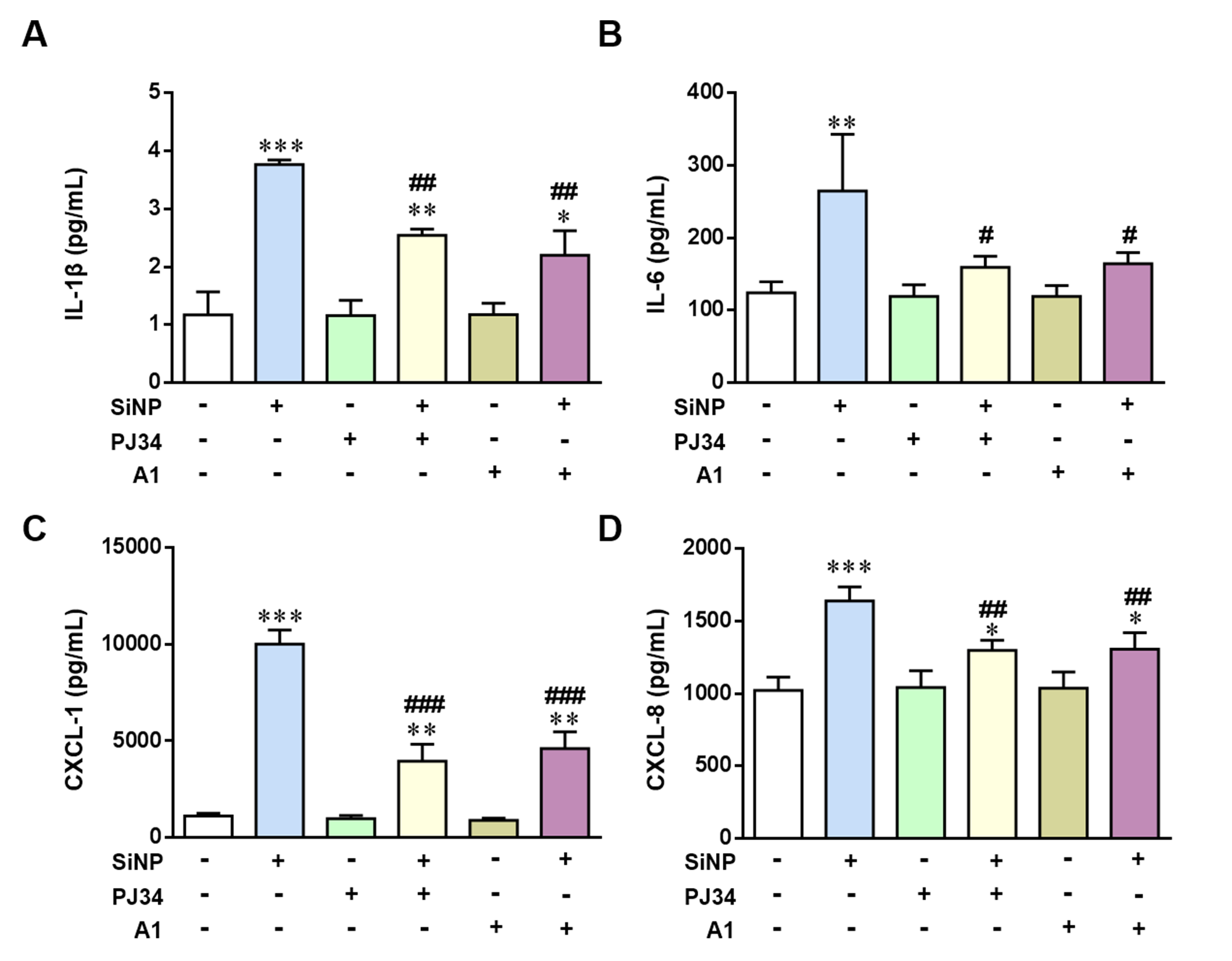

Supplement: Supplementary file 2 — Additional file 2 : Figure S2 Inhibition of PARP and TRPM2 channel reduces SiNPs-induced cytokines and chemokines genernation in BEAS-2B cells. IL-1β (A), IL-6 (B), CXCL-1 (C) and CXCL-8 (D). Cells were incubated with SiNPs (100 μg/mL) in the absence or presence of PJ34 or compound A1 (both at 10 μM). Data are presented as mean ± SD from three independent experiments. * P < 0.05, ** P < 0.01 compared with the control group. #P < 0.05, ##P < 0.01, ###P < 0.001 compared with SiNPs-treated group. [file 12989_2020_353_MOESM2_ESM.tif]

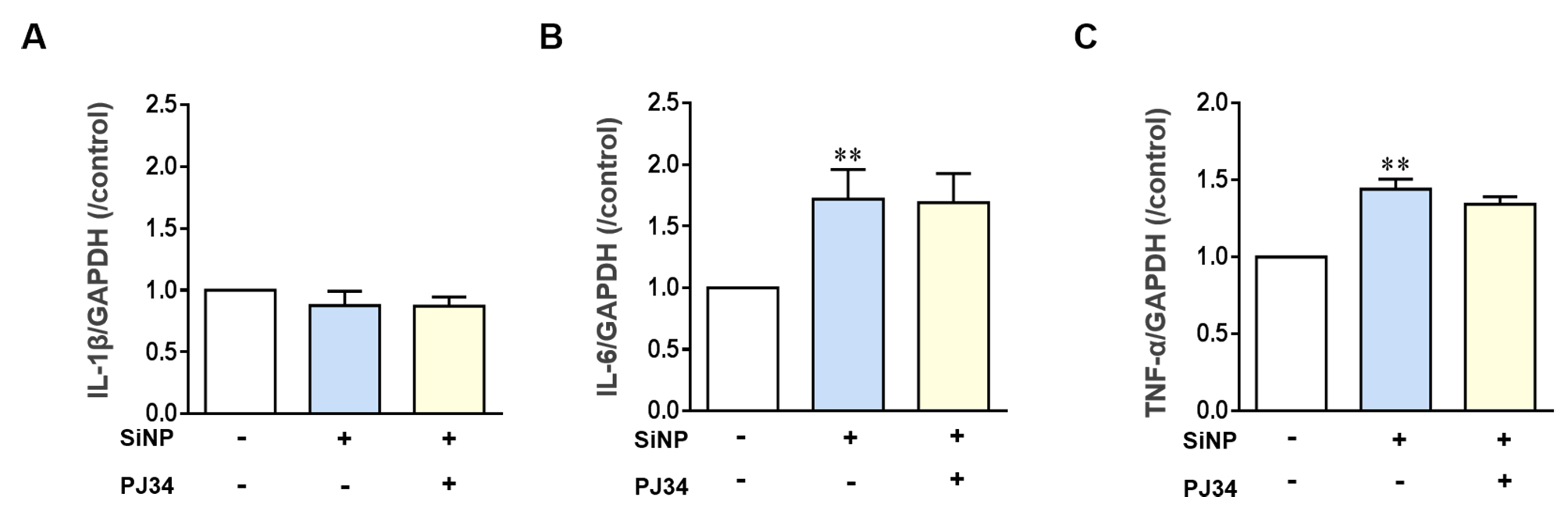

Supplement: Supplementary file 3 — Additional file 3 : Figure S3 Treatment with PJ34 has no effect on SiNPs-induced cytokines and chemokines generation in iBMDM cells. IL-1β (A), IL-6 (B), and TNF-α (C). Cells were exposed to SiNPs (100 μg/mL) in the absence or presence of PJ34 (10 μM). Data are presented as mean ± SD from three independent experiments. ** P < 0.01 compared with the control group. [file 12989_2020_353_MOESM3_ESM.tif]

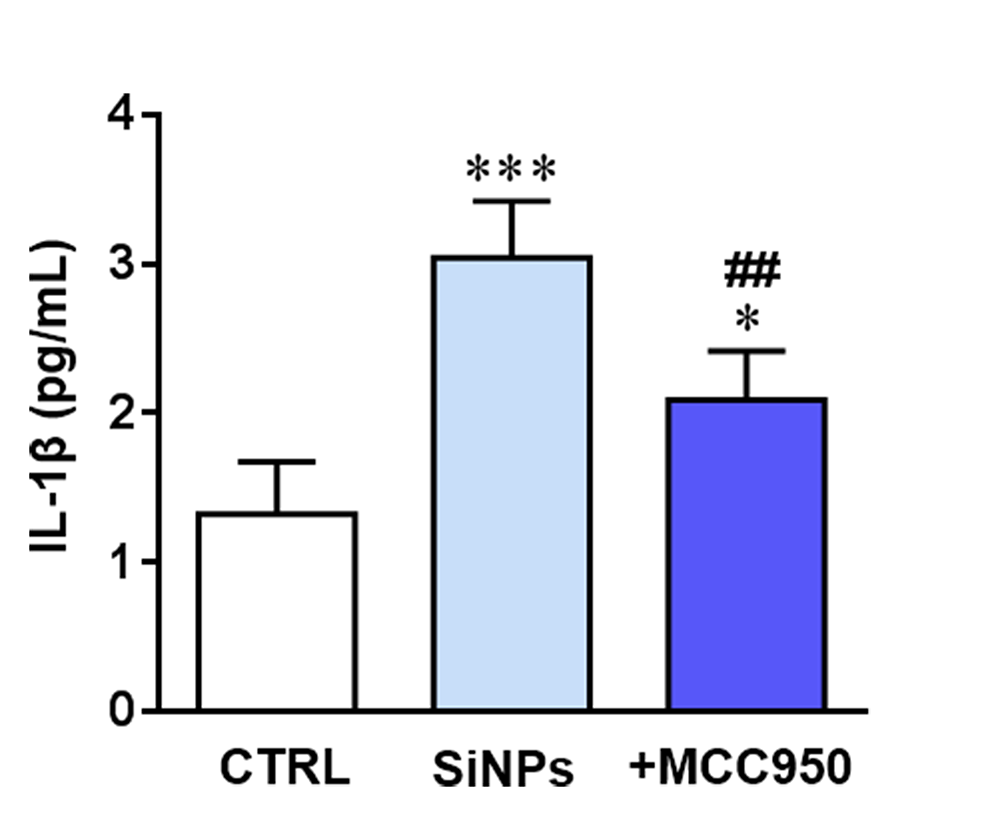

Supplement: Supplementary file 4 — Additional file 4 : Figure S4. Inhibition of NLRP3 inflammasome attenuates SiNPs-induced inflammation in BEAS-2B cells. Cells were co-incubated with SiNPs (100 μg/mL) for 24 h in the absence or presence of MCC950 (100 nM). Data are presented as mean ± SD from three independent experiments. * P < 0.05, *** P < 0.001 compared to the control group. ##P < 0.01 compared to SiNPs-treated group. [file 12989_2020_353_MOESM4_ESM.tif]

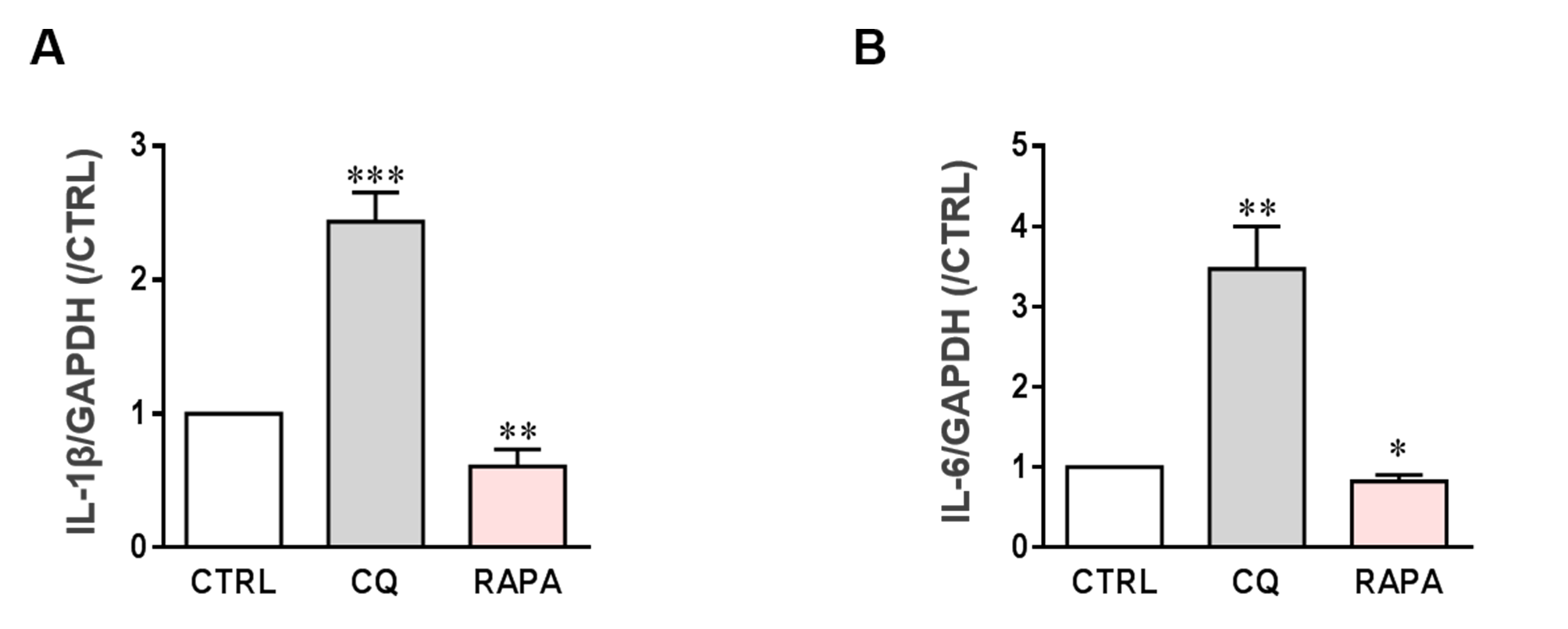

Supplement: Supplementary file 5 — Additional file 5 : Figure S5. Autophagic flux regulates inflammatory responses in BEAS-2B cells. IL-1β (A) and IL-6 (B). BEAS-2B cells were treated with 50 μM chloroquine (CQ) or 100 nM rapamycin (RAPA) for 3 h. Data are presented as mean ± SD from three independent experiments. * P < 0.05, ** P < 0.01, *** P < 0.001 compared to the control group. [file 12989_2020_353_MOESM5_ESM.tif]

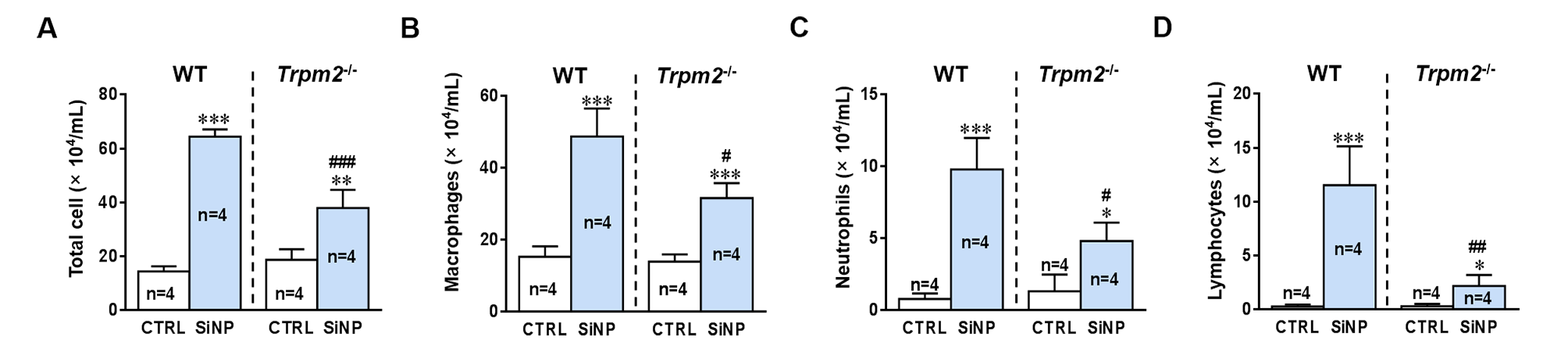

Supplement: Supplementary file 6 — Additional file 6 : Figure S6. Analysis of inflammatory cells in the BALFs of WT and Trpm2−/− mice. The counts of total cells (A), macrophages (B), neutrophils (C), lymphocytes (D) in the BALFs from WT and Trpm2−/− mice after i.t instillation of SiNPs (10 mg/kg). Data are mean ± SD from 8 mice. *P < 0.05, **P < 0.01, ***P < 0.001 compared with the control group. #P < 0.05, ##P < 0.01 compared with the WT mice. [file 12989_2020_353_MOESM6_ESM.tif]
